# Unraveling the potential mechanisms of the anti-osteoporotic effects of the *Achyranthes bidentata–Dipsacus asper* herb pair: a network pharmacology and experimental study

**DOI:** 10.3389/fphar.2023.1242194

**Published:** 2023-10-02

**Authors:** Tao Li, Wenzhao Li, Xiaoning Guo, Tingting Tan, Cheng Xiang, Zhengxiao Ouyang

**Affiliations:** ^1^ Department of Orthopedics, The Second Xiangya Hospital, Central South University, Changsha, Hunan, China; ^2^ Department of Immunology, School of Basic Medical Science, Central South University, Changsha, Hunan, China

**Keywords:** osteoporosis, *Achyranthes bidentata*, *Dipsacus asper*, network pharmacology, MAPK

## Abstract

**Background:** Osteoporosis is a prevalent bone metabolism disease characterized by a reduction in bone density, leading to several complications that significantly affect patients’ quality of life. The Achyranthes bidentata–Dipsacus asper (AB–DA) herb pair is commonly used in Traditional Chinese Medicine (TCM) to treat osteoporosis. This study aimed to investigate the therapeutic compounds and potential mechanisms of AB–DA using network pharmacology, molecular docking, molecular dynamics simulation, and experimental verification.

**Methods:** Identified compounds of AB–DA were collected from the Traditional Chinese Medicine Systems Pharmacology Database and Analysis Platform (TCMSP), Traditional Chinese Medicine Information Database (TCM-ID), TCM@Taiwan Database, BATMAN-TCM, and relevant literature. The main bioactive ingredients were screened based on the criteria of “OB (oral bioavailability) ≥ 30, DL (drug-likeness) ≥ 0.18.” Potential targets were predicted using the PharmMapper and SwissTargetPrediction websites, while disease (osteoporosis)-related targets were obtained from the GeneCards, DisGeNET, and OMIM databases. The PPI network and KEGG/GO enrichment analysis were utilized for core targets and pathway screening in the STRING and Metascape databases, respectively. A drug–compound–target–pathway–disease network was constructed using Cytoscape software to display core regulatory mechanisms. Molecular docking and dynamics simulation techniques explored the binding reliability and stability between core compounds and targets. *In vitro* and *in vivo* validation experiments were utilized to explore the anti-osteoporosis efficiency and mechanism of sitogluside.

**Results:** A total of 31 compounds with 83 potential targets for AB–DA against osteoporosis were obtained. The PPI analysis revealed several hub targets, including AKT1, CASP3, EGFR, IGF1, MAPK1, MAPK8, and MAPK14. GO/KEGG analysis indicated that the MAPK cascade (ERK/JNK/p38) is the main pathway involved in treating osteoporosis. The D–C–T–P–T network demonstrated therapeutic compounds that mainly consisted of iridoids, steroids, and flavonoids, such as sitogluside, loganic acid, and β-ecdysterone. Molecular docking and dynamics simulation analyses confirmed strong binding affinity and stability between core compounds and targets. Additionally, the validation experiments showed preliminary evidence of antiosteoporosis effects.

**Conclusion:** This study identified iridoids, steroids, and flavonoids as the main therapeutic compounds of AB–DA in treating osteoporosis. The underlying mechanisms may involve targeting core MAPK cascade (ERK/JNK/p38) targets, such as MAPK1, MAPK8, and MAPK14. *In vivo* experiments preliminarily validated the anti-osteoporosis effect of sitogluside. Further in-depth experimental studies are required to validate the therapeutic value of AB–DA for treating osteoporosis in clinical practice.

## 1 Introduction

Osteoporosis is a prevalent bone metabolism disease that affects over 200 million people worldwide (McDonald et al., 2021; Grewe et al., 2022). It is characterized by a reduction in bone density, which greatly increases the risk of fractures (NIH Consensus Development Panel on Osteoporosis Prevention et al., 2001). Osteoporotic fractures and associated complications can have a significant and lasting impact on patients’ quality of life, sometimes even threatening their lives, and place a considerable cost on society and individuals (Compston et al., 2019; Liang et al., 2022). With the aging of the global population, osteoporosis has become a pressing health problem (Jiang et al., 2020).

Anti-osteoporosis drugs such as estrogen, raloxifene, bisphosphonates, calcitonin, and parathyroid hormone (PTH) are commonly used in clinical practice (Chen et al., 2022). These medications, however, have side effects and severe responses that restrict their long-term usage. For example, bisphosphonates may cause jaw osteonecrosis and renal impairment (Li et al., 2021). There is, therefore, an urgent need to identify potential anti-osteoporosis drugs that are both more effective and safer.

Traditional Chinese Medicine (TCM) has a rich history and has been widely used in Asia to treat various diseases, including osteoporosis (Huang et al., 2022). TCM is cost-effective and has fewer side effects than chemosynthetic drugs, making it more suitable for long-term use (Mukwaya et al., 2014). The application of TCM in modern society provides a new pathway for complementary and alternative medicine (CAM) treatment (Chu et al., 2022). Over the years, many TCM treatments and prescriptions have been used to treat various orthopedic diseases, especially osteoporosis and fractures, with great success (Suvarna et al., 2018; Peng et al., 2022). TCM’s prescription to treat osteoporosis can also play a comprehensive role in regulating body function and relieving pain (Feng et al., 2022). A Chinese herb pair, generally composed of two kinds of herbal medicine, is the essence of TCM prescriptions. Compared with all herbs in prescriptions, studying and elucidating the complex pharmacological mechanism of herb pairs is simpler and more beneficial (Liu et al., 2020).

*Achyranthes bidentata* (AB), also known as Niu Xi, is included in the Chinese, Japanese, and Korean pharmacopoeia (He et al., 2017). Additionally, its dried roots are regularly used in TCM for osteoporosis (Yan et al., 2019). A number of biological activities, including anti-osteoporosis (He et al., 2010; Zhang et al., 2012; Jiang et al., 2014; Suh et al., 2014; Zhang M. et al., 2018; Zhang S. et al., 2018), anti-tumor (Jin et al., 2007), and anti-oxidant (Huang et al., 2015), have been demonstrated by contemporary pharmacological research on AB extracts. *Dipsacus asper* (DA), also known in Chinese as Xu Duan —meaning “to rebuild fractures and unite bones”—is discussed in Shennong’s Classic of Material Medicine, which is the earliest source (Tao et al., 2020). DA can be used to treat muscle pain and bone repair, golden sores, and collapses (Wu et al., 2022). According to modern pharmacological investigations, numerous disorders, including osteoporosis and osteoarthritis, have been successfully treated with DA (Liu et al., 2019; Yu et al., 2019; Zhang et al., 2019). Jiegudan capsules, which contain AB and DA, are a common traditional Chinese medicine prescription to treat osteoporosis. Although many compounds have been isolated from AB and DA, the potential pharmacological mechanisms of AB–DA herb pairs and their interactions with osteoporosis-related targets and pathways remain unclear and need further exploration.

**FIGURE 1:**
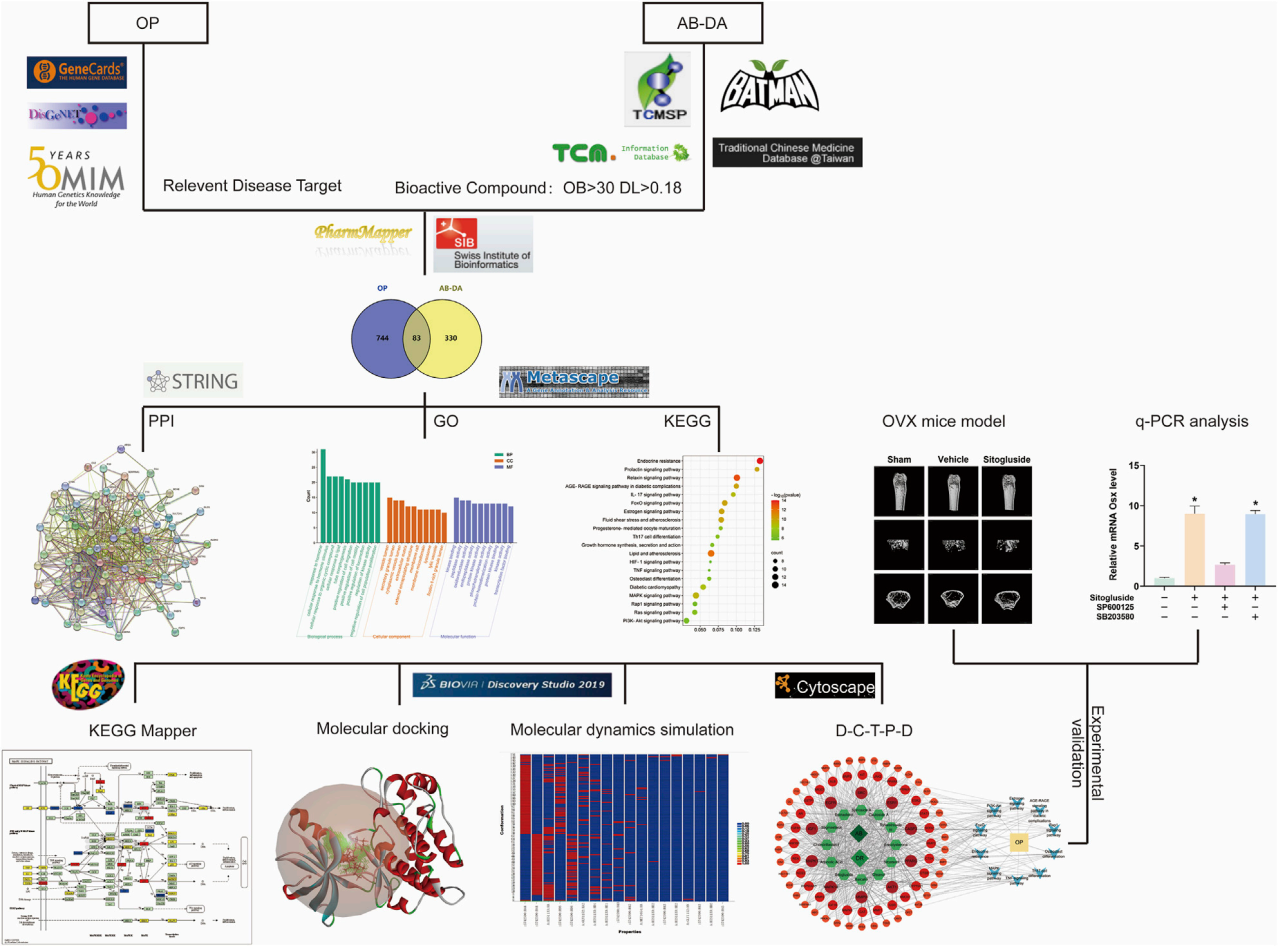
Study flow chart to investigate the potential underlying mechanisms for AB–DA treatment of osteoporosis.

In recent years, the use of network pharmacology has become increasingly popular for exploring the interaction network of TCM therapy (Shuai et al., 2020). Molecular docking, a virtual screening technology that simulates the behavior of small-molecule ligands at the binding sites of receptor proteins, has also gained popularity for developing novel drugs (Pagadala et al., 2017). This research aims to elucidate the potential mechanism in TCM of the AB–DA herb pair for treating osteoporosis, bioinformatics prediction by network pharmacology, molecular docking, and molecular dynamics simulation, and verify these via alkaline phosphatase (ALP) activity, osteoblast mineralization assays, Western blot, q-PCR, and an ovariectomy (OVX) osteoporosis mouse model. A flow chart outlining the study’s approach is presented in Figure 1.

## 2 Materials and methods

### 2.1 Screening of bioactive compounds of AB–DA

The TCM@Taiwan (http://tcm.cmu.edu.tw/zh-tw/) Database (Chen, 2011), Traditional Chinese Medicine Systems Pharmacology Database and Analysis Platform (TCMSP http://lsp.nwu.edu.cn/tcmsp.php) (Ru et al., 2014), BATMAN-TCM (L et al., 2016) (a bioinformatics analytical tool for the molecular mechanisms of TCM: http://bionet.ncpsb.org.cn/), Traditional Chinese Medicine Information Database (Kang et al., 2013) (TCM-ID http://bidd.group), and relevant literature were utilized to acquire all identified AB–DA compounds. ADME (absorption, distribution, metabolism, and excretion) properties were applied to screen bioactive ingredients, and the screening criteria were set as “oral bioavailability (OB)≥ 30, drug-likeness (DL)≥ 0.18” (Gao et al., 2022) to screen the compounds from the TCMSP database. Similarly, compounds from different sources were screened in the SwissADME (Daina et al., 2017) database using their pharmacokinetic properties (http://www.swissadme.ch).

### 2.2 Relevant targets of AB–DA compound and osteoporosis

To predict potential targets based on their spatial configuration, the compounds generated in the previous step were imported into the SwissTargetPrediction (Daina et al., 2019) (http://www.swisstargetprediction.ch/) and PharmMapper databases (Liu et al., 2010) (http://www.lilab-ecust.cn/pharmmapper/). The UniProt ID of the target was converted into a standardized gene name using the UniProt database (Holzhüter and Geertsma, 2022) (https://www.uniprot.org). The keyword “osteoporosis” was searched in the GeneCards (Barshir et al., 2021) (https://www.genecards.org/), DisGeNET (Piñero et al., 2020) (https://www.disgenet.org), and Online Mendelian Inheritance in Man (Li et al., 2012) (OMIM, https://omim.org) databases to obtain relevant targets. Then, the overlapping targets identified by Venn diagram were considered as targets of AB–DA for the treatment of osteoporosis after merging and removing duplicates.

### 2.3 PPI network for core target selection

To identify potential hub genes, a protein–protein interaction (PPI) network was established in the STRING (Szklarczyk et al., 2021) (http://string-db.org, Version 11.5) database, with a focus on the anti-osteoporosis efficacy of AB–DA in *Homo sapiens* and an interaction score threshold of 0.4. Topological analysis was performed, and the core targets of AB–DA for treating osteoporosis were accurately selected by using the Cytoscape plug-ins MCODE (molecular complex detection) and CytoHubba (Ye et al., 2022).

### 2.4 GO and KEGG pathway enrichment analyses for the core pathways

Analysis of Gene Ontology (GO) functions, including cellular component (CC), molecular function (MF), and biological process (BP), and Kyoto Encyclopedia of Genome and Genome (KEGG) pathway enrichment analysis, was utilized to clarify the key anti-osteoporosis mechanism of the AB–DA herb pair. When entering the targets into the Metascape database (http://www.metascape.org/), the cut-off *p*-value, minimum overlap value, and concentration value were set to 0.01, 3, and 1.5, respectively (Zhou et al., 2019). False-positive rate (FPR) analysis was eliminated using the Benjamini–Hochberg method with a q-value of 0.05 or lower (Zou et al., 2016). The enriched findings were displayed as bar and bubble plots on the bioinformatics website using the R package (http://www.bioinformatics.com.cn/). Comprehensive information on the most significantly enriched pathway was then extracted and colored (Kanehisa and Sato, 2020). Finally, a herb–compound–target–pathway–disease network was created using Cytoscape software (v.3.9.1, https://cytoscape.org/) to present the complicated network of the AB–DA herb pair in the treatment of osteoporosis.

### 2.5 Molecular docking to validate binding affinity

The SwissDock platform (Grosdidier et al., 2011) (http://www.swissdock.ch/) is an online molecular docking (MD) tool to determine the binding affinity from each binding site between small molecule ligands and receptor proteins. The X-ray diffraction of the protein crystal structure of key targets were downloaded from the Protein Data Bank (PDB) database (www.rcsb.org) (Nakamura et al., 2022). The binding sites were ranked based on their binding affinity scores, with the site having the smallest score considered the best binding site. Discovery Studio 2019 software (https://www.3ds.com) was used to visualize the binding details (Sultana et al., 2022).

### 2.6 Molecular dynamics simulation to validate binding stability

To investigate the stability of the complexes between small-molecule ligands and proteins, molecular dynamics simulations (MDS) were performed using the Standard Dynamics Cascade subunit of the Discovery Studio 2019 software. The ligand–protein complex with the lowest binding affinity score according to molecular docking analysis was selected (Hu et al., 2022). In this simulation system, water molecules are used to fill the solvent chamber, and Cl and Na+ ions are used to maintain an electrically neutral state. The simulation time was set as 300 ps, and the heating, balancing, and manufacturing phases were carried out after the system was balanced by an NPT ensemble, which fixed the pressure, temperature, and particle number. The analysis of the locus was performed using root mean square fluctuation (RMSF), root mean square deviation (RMSD), and hydrogen bond properties to produce the results.

### 2.7 Reagent and cell culture

Sitogluside, identified as one of the most promising bioactive compounds in the AB–DA herb pair, was further investigated for its anti-osteoporotic effects and associated mechanisms. Sitogluside was procured from the Dalian Meilunbio company and solubilized in dimethyl sulfoxide (DMSO). Human fetal osteoblast (hFOB) cells were obtained from the American Type Culture Collection (ATCC) and cultured in six-well plates using Dulbecco’s Modified Eagle Medium (DMEM) supplemented with 10% fetal bovine serum (FBS, Gibco, United States), 1% penicillin–streptomycin (P/S), and 0.3 mg/mL Geneticin (G418). The JNK-specific inhibitor (SP600125) and p38-specific inhibitor (SB203580) were acquired from Absin (Shanghai). The cells were maintained in a humidified sterile atmosphere at 34 °C. They were subsequently treated with sitogluside when they reached a confluence of 60%–80% per well.

### 2.8 Alkaline phosphatase activity, Alizarin red staining mineralization, and osteoclast differentiation assays

For alkaline phosphatase (ALP) activity analysis, the BCIP/NBT Alkaline Phosphatase color development kit (Beyotime Institute of Biotechnology, Shanghai, China) was used according to the manufacturer’s procedure. Briefly, osteoblast precursor cells were seeded at 3 × 10^4^ cells/well in 24-well plates and grown for 14 days in osteogenic media (DMEM +10% FBS +1% P/S +100 M ascorbic acid +2 mM 2-glycerophosphate +10 nM dexamethasone). The stained culture plates were photographed using a microscope (Leica image analysis system, Q500MC) and quantified using ImageJ software (National Institutes of Health, Bethesda, MD, United States). In addition, a 2% ARS reagent (Beyotime Institute of Biotechnology) was used to detect matrix mineralization, with the same protocol as ALP assay except the dye. In addition, we also investigated the effect of sitogluside on the osteoclast to fully illustrate the anti-OP effects. The RAW264.7 (osteoclast precursor) cells were treated with a different concentration of sitogluside and supplemented with 50 ng/mL receptor activator of NF-κB ligand (RANKL) as an osteoclast formation stimulator. Cells were then fixed in paraformaldehyde and stained with tartrate-resistant acid phosphatase (TRAP) after the intervention.

### 2.9 Ovariectomized mouse model

This study received ethical approval from the Animal Care Committee of the Second Xiangya Hospital of Central South University. A total of 30 10-week-old female C57BL/6 mice were procured from SLAC Laboratory Animal Co. Ltd. (SLACCAS, Shanghai, China). They were acclimatized in specific pathogen-free (SPF) cages for 1 week, during which measures were taken to minimize animal suffering through the use of anesthesia and sterile techniques during the surgical procedures. Bilateral ovariectomy (OVX) or sham surgery (retroperitoneal incision without ovariectomy) was performed based on group assignment, as described in the following paragraph.

Following the surgical procedures, the mice were randomly assigned to one of three groups: sham group (non-OVX mice, *n* = 10), vehicle group (OVX mice, *n* = 10), and sitogluside group (OVX mice intraperitoneally injected with sitogluside at a dose of 10 mg/kg/day, *n* = 10).

After 12 weeks, all mice were euthanized by cervical dislocation, and the right femurs were dissected and fixed in 4% paraformaldehyde (PFA) for 48 h. High-resolution micro-computed tomography (μCT40, Scanco, Zurich, Switzerland) was employed for bone analysis at the following parameters: scanning voltage = 80 kV, electric current = 80 μA, and resolution = 10 μm. The relevant trabecular bone volume fractions (BV/TV), trabecular number (Tb. N), trabecular thickness (Tb. Th), and trabecular separation (Tb. Sp) were subsequently calculated to assess the protective efficacy of sitogluside in OVX mice. In addition, to investigate the biosafety of sitogluside in the OVX model, the main organs were also obtained and detected using hematoxylin-eosin (H&E) staining. The heart, liver, spleen, lung, and kidney were hence fixed with formalin and embedded in paraffin cut to a 4 μm section. They were dewaxed in xylene, rehydrated with concentration gradient ethanol, and then stained with H&E for histological examinations and morphometric analysis. The serum of the mice was also collected to examine the biomarkers of alanine aminotransferase (ALT), creatine kinase (CK), and blood urea nitrogen (BUN).

### 2.10 Quantitative real-time PCR analysis

Human osteoblast (hFOB) cells were treated with 40 μM sitogluside or combined with the specific inhibitor of JNK and p38, depending on the groups, with ascorbic acid added in the osteogenic media. The cells were then harvested using the RNeasy Mini kit (QIAGEN, CA, United States) to extract total RNA following the manufacturer’s protocol. Subsequently, cDNA synthesis was performed using the reverse transcriptase kit (Takara Biotechnology, Japan). Real-time PCR analysis was performed using the SYBR Premix Ex Taq kit (Takara Biotechnology, Japan). The PCR parameters were set as follows: 40 cycles (denaturation at 95 °C for 10 s and amplification at 60 °C for 30 s). The resultant data were recorded as cycle threshold (Ct) values, and the 2^−ΔΔCT^ method was employed for further analysis of RNA expression. In addition, to determine the modulated effect of sitogluside with JNK and osteogenic genes, the knockdown and activation of JNK expression were applied. ShRNA (shGnai3) was thus used to downregulate the expression level of JNK, and ASM was used as an activator to increase the p-JNK level (Meng et al., 2021). The alterations of osteogenic biomarkers were detected via q-PCR.

### 2.11 Western blot analysis

The hFOB cells were harvested by trypsin and then lysed in RIPA for 30 min on ice. Cell lysates were centrifuged at 12,000 g for 15 min at 4 C; the supernatant was collected, and protein content was quantified via the BSA protein assay kit following the manufacturer’s instruction. Proteins were separated by electrophoresis on 10%–12% SDS-PAGE at 100 V for 1.5 h and transferred onto a 0.45 μm polyvinylidene difluoride (PVDF) membrane at 250 mA for 1 h. The PVDF membrane was blocked with 5% non-fat milk in TBST buffer for 1 h at room temperature and incubated with primary antibody at 4 °C overnight. They were then incubated with secondary antibody for 1 h at room temperature and detected using the Chemiluminescence Kit.

### 2.12 Statistical analysis

Statistical analyses were conducted using GraphPad Prism 8.0.2 software (San Diego, United States). The data are presented as means ± standard deviation (SD). Data comparisons were performed using one-way analysis of variance (ANOVA), and statistical significance was determined by a *p*-value of less than 0.05.

## 3 Results

### 3.1 Relevant targets of AB–DA compound and osteoporosis

**FIGURE 2:**
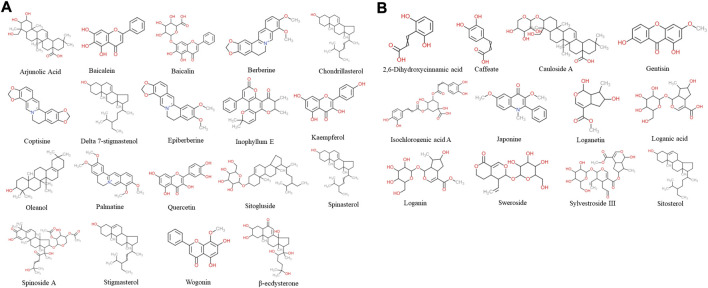
Chemical structure of 31 screened bioactive compounds. **(A)** Chemical structure of 19 bioactive compounds derived from AB. **(B)** Chemical structure of 12 bioactive compounds derived from DA; sitosterol is common to both.

**FIGURE 3:**
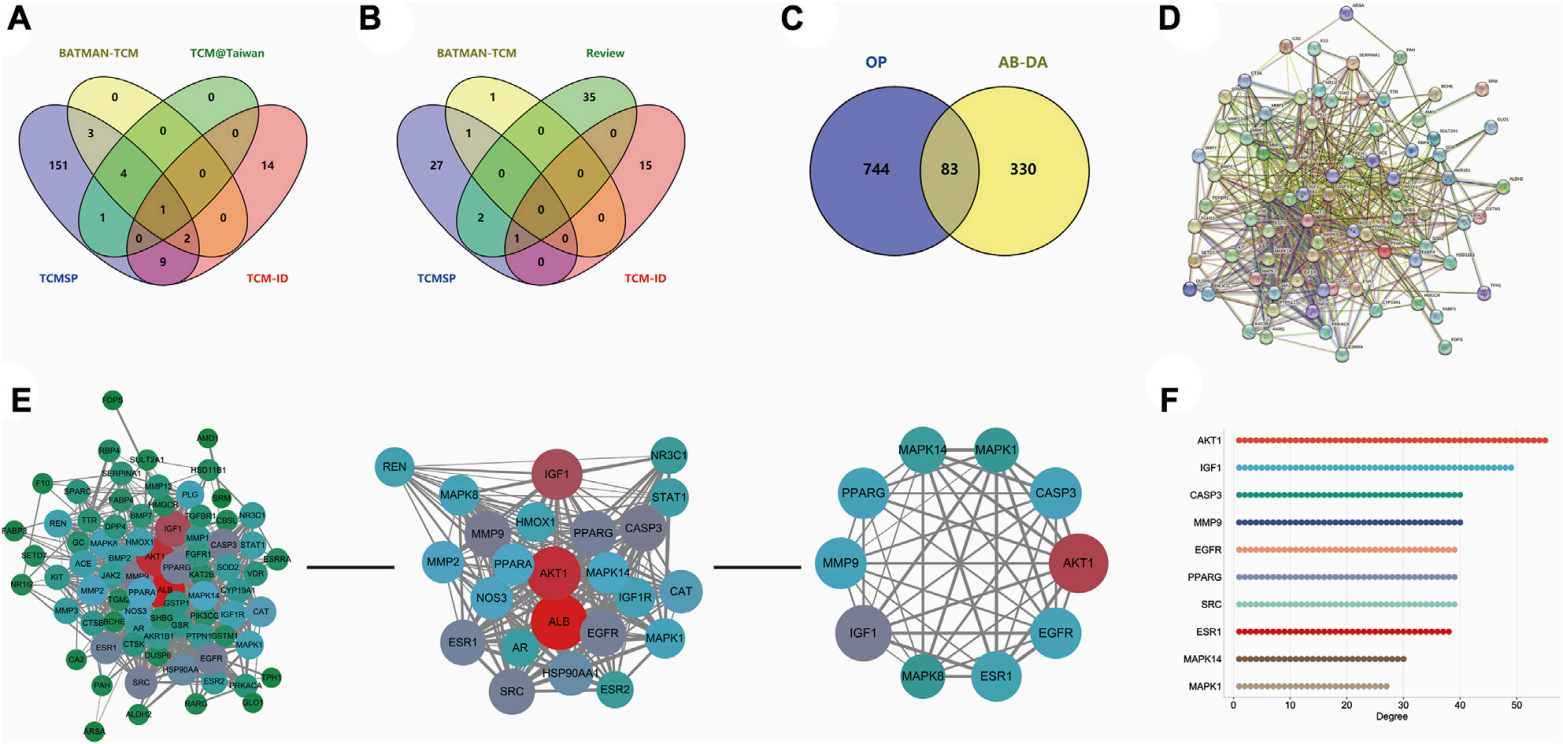
Data collection and hub gene screening for AB–DA against osteoporosis. **(A)** Venn diagram of the identified compounds of AB; data derived from TCMSP, TCM@Taiwan, BATMAN-TCM, and TCM-ID databases. **(B)** Venn diagram of the identified compounds of DA; data derived from the TCMSP, TCM-ID, and BATMAN-TCM databases and relevant literature. **(C)** Overlapping targets of AB–DA and osteoporosis, representing potential therapeutic targets. **(D)** PPI network analysis applied in the STRING database. **(E)** Plug-ins MCODE and CytoHubba of Cytoscape software to screen hub genes. **(F)** Degree value of top 10 hub genes.

According to TCM databases, 185 components of AB and 82 compounds of DA were obtained in this work (Figures 3A, B). After filtration by screening criteria, 19 potential bioactive compounds of AB were obtained: arjunolic acid, baicalein, baicalin, berberine, chondrillasterol, coptisine, delta-7-stigmastenol, epiberberine, inophyllum E, kaempferol, oleanol, palmatine, quercetin, sitogluside, spinasterol, spinoside A, stigmasterol, wogonin, and β-ecdysterone. The 12 compounds from DA were 2,6-dihydroxycinnamic acid, caffeate, cauloside A, gentisin, isochlorogenic acid A, japonine, loganetin, loganic acid, loganin, sweroside, and sylvestroside III. Sitosterol is a common compound of both AB and DA (details shown in Supplementary Table S1; structures shown in Figure 2).

After merging and duplicating results, 413 potential targets associated with 31 bioactive compounds were obtained, and 827 relevant targets of osteoporosis were acquired. Some 83 overlapped genes on Venn between AB–DA ingredients and osteoporosis were regarded as potential therapeutic targets (Figure 3C; details shown in Supplementary Table S2).

### 3.2 PPI network of AB–DA against osteoporosis

A total of 83 targets of AB–DA against osteoporosis were imported to the STRING database; after deleting the disconnect targets, a PPI network with 78 nodes and 702 edges was constructed. Cytoscape software was utilized for further visualization, and plug-ins of MCODE and CytoHubba based on the topological parameters were applied to screen the hub genes, including AKT1, IGF1, CASP3, MMP9, EGFR, PPARG, ESR1, MAPK1, MAPK8, and MAPK14 (Figures 3D–F).

### 3.3 GO and KEGG pathway enrichment analyses of AB–DA against osteoporosis

**FIGURE 4:**
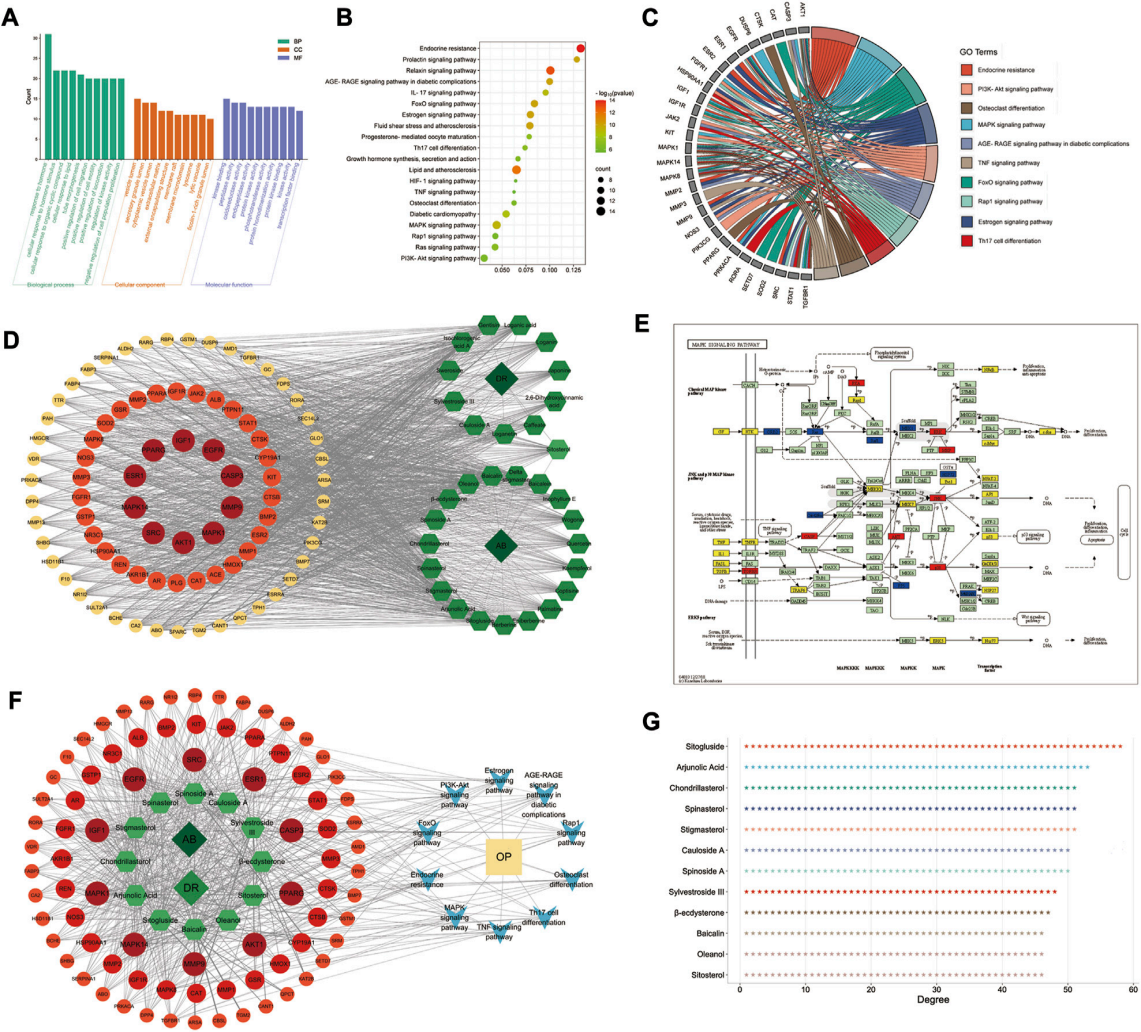
GO/KEGG enrichment analysis and D–C–T–P–D network of AB–DA in the treatment of osteoporosis. **(A)** Top 10 enriched GO items; left to right are biological process (BP), cellular compound (CC), and molecular function (MF), respectively. **(B)** Bubble diagram of top 20 enriched KEGG pathways. **(C)** GO chord chart presenting the corresponding relationship between core targets and pathways. **(D)** D–C–T network map; dark green diamond represents drugs (AB and DA), light green hexagon represents 31 bioactive compounds of AB–DA, and circles colored orange to red represent targets with low to high degrees. **(E)** MAPK signaling pathway mapped and colored by KEGG Mapper database; therapeutic targets of AB–DA are shown in red, targets of AB–DA but without therapeutic effect on osteoporosis are shown in blue, and other targets of osteoporosis are shown in yellow. **(F)** D–C–T–P–D network exhibits the regulatory mechanisms for AB–DA in the treatment of osteoporosis; dark green diamond represents drugs (AB and DA), light green hexagon represents compounds of AB–DA, circles colored orange to red represent targets with low to high degrees, blue V icon represents enriched core pathways, and yellow rectangle indicates the disease (osteoporosis). **(G)** the degree values of top 12 bioactive compounds.

GO and KEGG pathway enrichment analyses were performed in the Metascape database, and 83 targets with 875 GO items were enriched. It contained 772 biological processes (BP), 29 cellular components (CC), and 74 molecular functions (MF) items (*p* < 0.01, adjusted q < 0.05) (details shown in Figure 4A).

A total of 135 KEGG pathways were significantly enriched, which mainly included the MAPK signaling pathway, osteoclast differentiation, PI3K-Akt signaling pathway, and endocrine resistance (Figures 4B, C). We further mapped and colored the regulation details of the MAPK cascade in the KEGG mapper database; in that map, red objects represent targets of AB–DA against osteoporosis, blue objects show the targets of AB–DA without the therapeutic effects of osteoporosis, and the other untargeted targets of osteoporosis are colored yellow (Figure 4E).

### 3.4 Drug–compound–target–pathway–disease network analysis

A drug–compound–target network was constructed using Cytoscape to illustrate core compounds and targets (Figure 4D); the core bioactive compounds included sitogluside, arjunolic acid, chondrillasterol, stigmasterol, spinasterol, spinoside A, cauloside A, sylvestroside III, β-ecdysterone, sitosterol, oleanol, and baicalin (ranked by degree value) (Figure 4G). A drug–compound–target–pathway–disease (D–C–T–P–D) network was then constructed to exhibit the complex molecular mechanisms of AB–DA anti-osteoporosis with multi-compound, multi-target, and multi-pathway characteristics. The dark green diamond represents the drugs (AB and DA), the light green hexagon represents the compounds of AB–DA, the circles colored orange to red represent targets with low to high degrees, the blue V icon represents the core enriched pathways, and the yellow rectangle indicates the disease (osteoporosis) (Figure 4F).

### 3.5 Molecular docking

**FIGURE 5:**
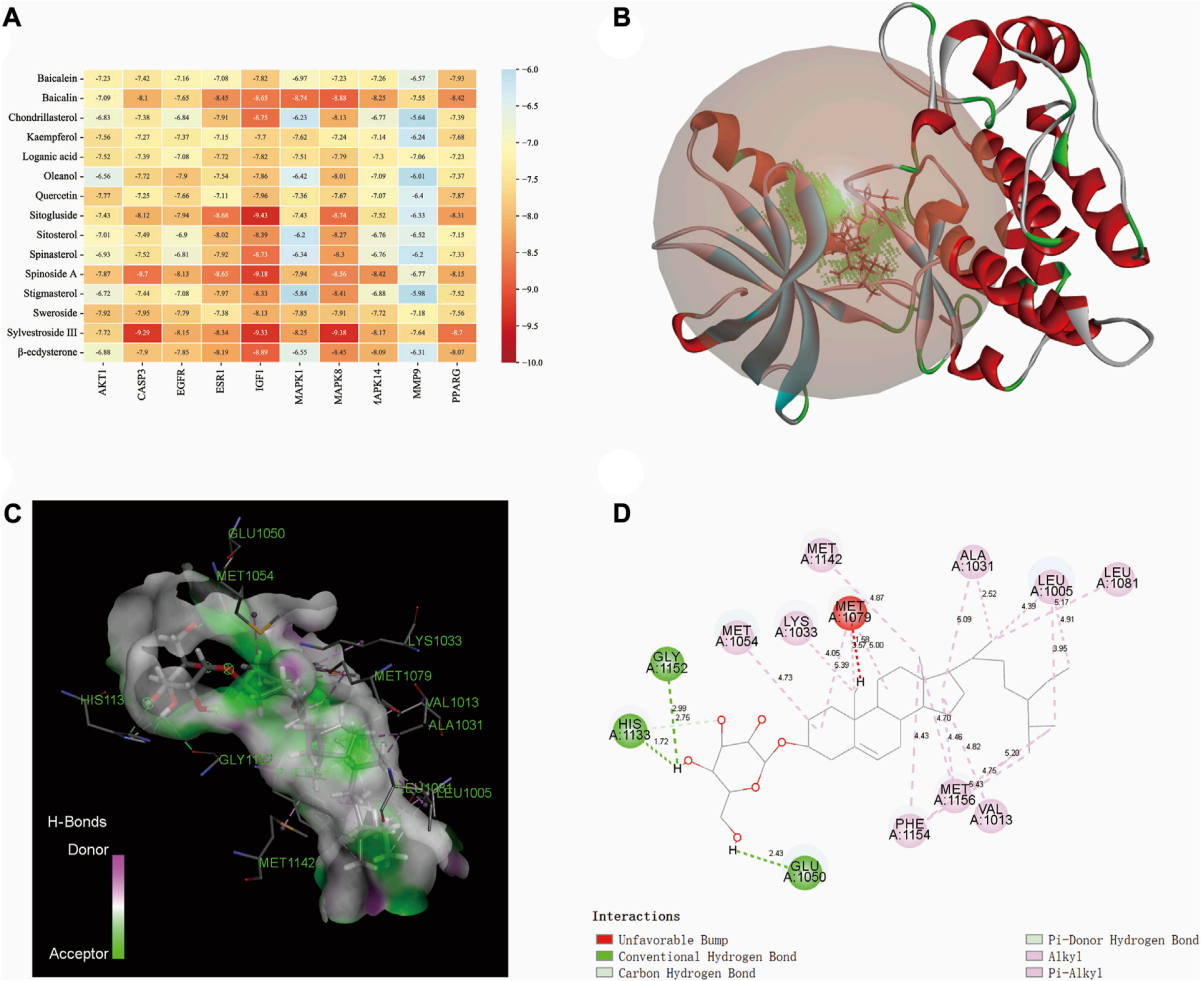
Molecular docking between bioactive compounds and core targets. **(A)** Binding affinity heatmap of compound ligand–protein receptor complexes, showing stronger binding affinity. **(B)** Binding details of the sitogluside–IGF1 complex (3D). **(C)** Binding details of the sitogluside–IGF1 complex (spatial structure). **(D)** Binding details of intermolecular force types of the sitogluside–IGF1 complex (2D).

The binding affinity between the core compounds and core targets are shown by heatmap (Figure 5A). According to relevant theories of molecular docking, the results of binding affinity < −5.0 kcal/mol suggest that there is a good spontaneous binding activity between molecule ligands and protein receptors, and results < −7.0 kcal/mol are stronger. Our research results showed that all the compounds had good binding activity with core targets, with binding affinities ranging from −5.64 kcal/mol to −9.43 kcal/mol. Sitogluside has the highest binding activity with IGF1. As shown in Figures 5B–D, the molecular interaction forces between IGF1 and sitogluside include π–donor hydrogen bond, π–alkyl bond, conventional hydrogen bond, carbon–hydrogen bond, and alkyl bond. The distances between the sitogluside atoms and amino acid residues of IGF1 range from 1.72 Å (number 1133, histidine residue) to 5.43 Å (number 1154, phenylalanine residue).

### 3.6 Molecular dynamics simulation

**FIGURE 6:**
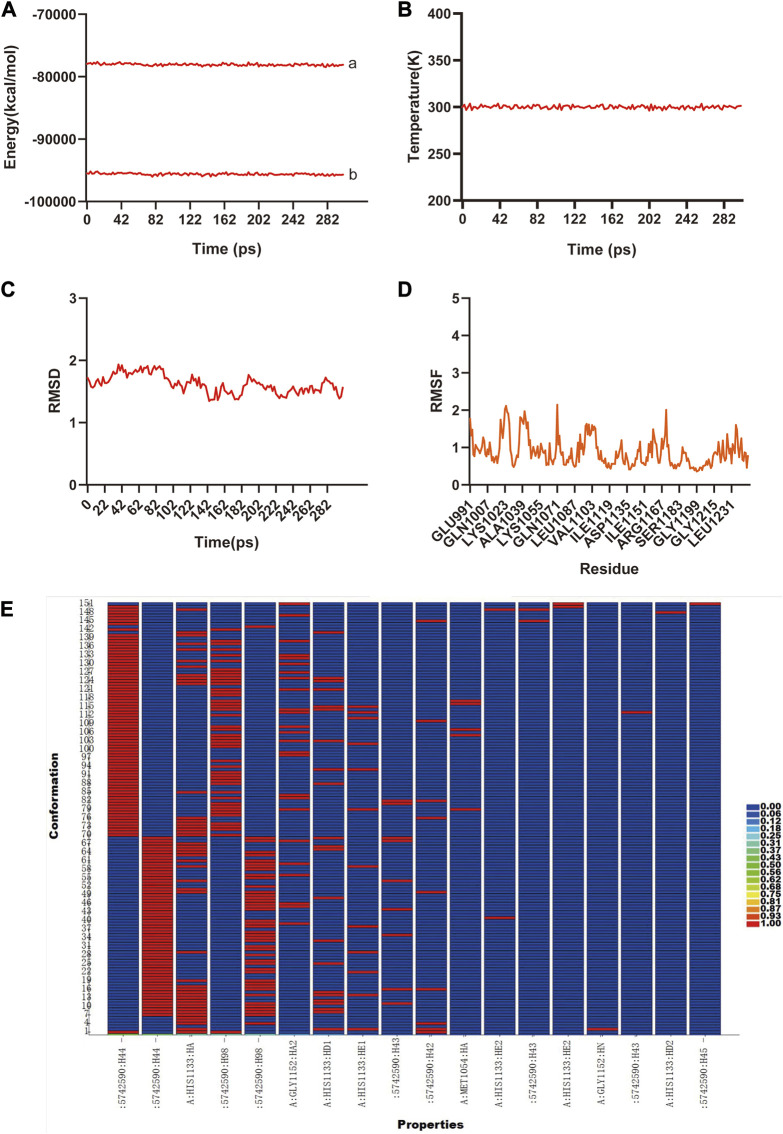
The molecular dynamic simulations to calculate the binding stability of sitogluside–IGF1 complex. **(A)**, The total energy (a) and potential energy (b) curves of the whole ensemble in 300 ps simulation, showed the stability. **(B)**, The temperature alteration of whole ensemble is controllable in 300 ps simulation. **(C)**, The RMSD curve present the conformational alternation of receptor made by ligand. **(D)**, The RMSF curve showed the conformational alternation of amino acid residues. **(E)**, The hydrogen bond heat map of sitogluside–IGF1 complex.

The previous analysis of molecular docking showed the strong binding affinity between AB–DA compounds and core targets, and molecular dynamics simulation was utilized to identify the stability of the ligand–protein complex after docking by conformation alternation with potential energy under Newton’s law of motion. After the 300 ps simulation, the energy and temperature alternate tendency of the ensemble, hydrogen bond, RMSD, and RMSF changes of ligand–receptor interaction were calculated for stability analysis. RMSD was used to analyze the conformational alternation of receptors made by the ligand, and the results showed that curves and fluctuations only occurred at the beginning of the 80 ps simulation, and then tended to be stable (Figure 6A). The RMSF curve was utilized to monitor the conformational alternation of amino acid residues, and results show that the whole process is stable with only some small random fluctuations—which also reflects the whole ensemble’s stability to some extent (Figure 6B). The same tendency is also observed in the hydrogen bond heatmap (Figure 6C). The energies and temperature alternation of the whole ensemble were also stable and controlled. Therefore, the overall results exhibited good stability between the small-molecule ligand–protein receptor complex.

### 3.7 Sitogluside promotes mineralization and ALP activity in osteoblast without any effect on osteoclast

To further investigate the impact of sitogluside on alkaline phosphatase (ALP) activity and mineralization in osteoblasts, we conducted ALP activity and ARS experiments. As illustrated in Supplementary Figure S3D, the sitogluside group exhibited increased ALP activity and mineralization compared to the control group. However, when shGnai3 intervention was introduced, the stimulatory effect of sitogluside on ALP activity and mineralization in osteoblasts was partially diminished. Subsequently, with the addition of the p-JNK activator ASM, the promotion of ALP activity and mineralization was restored. Additionally, our results indicated that sitogluside might not have a significant effect on osteoclast formation (Supplementary Figure S2).

### 3.8 Sitogluside administration protects against OVX-induced bone loss

**FIGURE 7:**
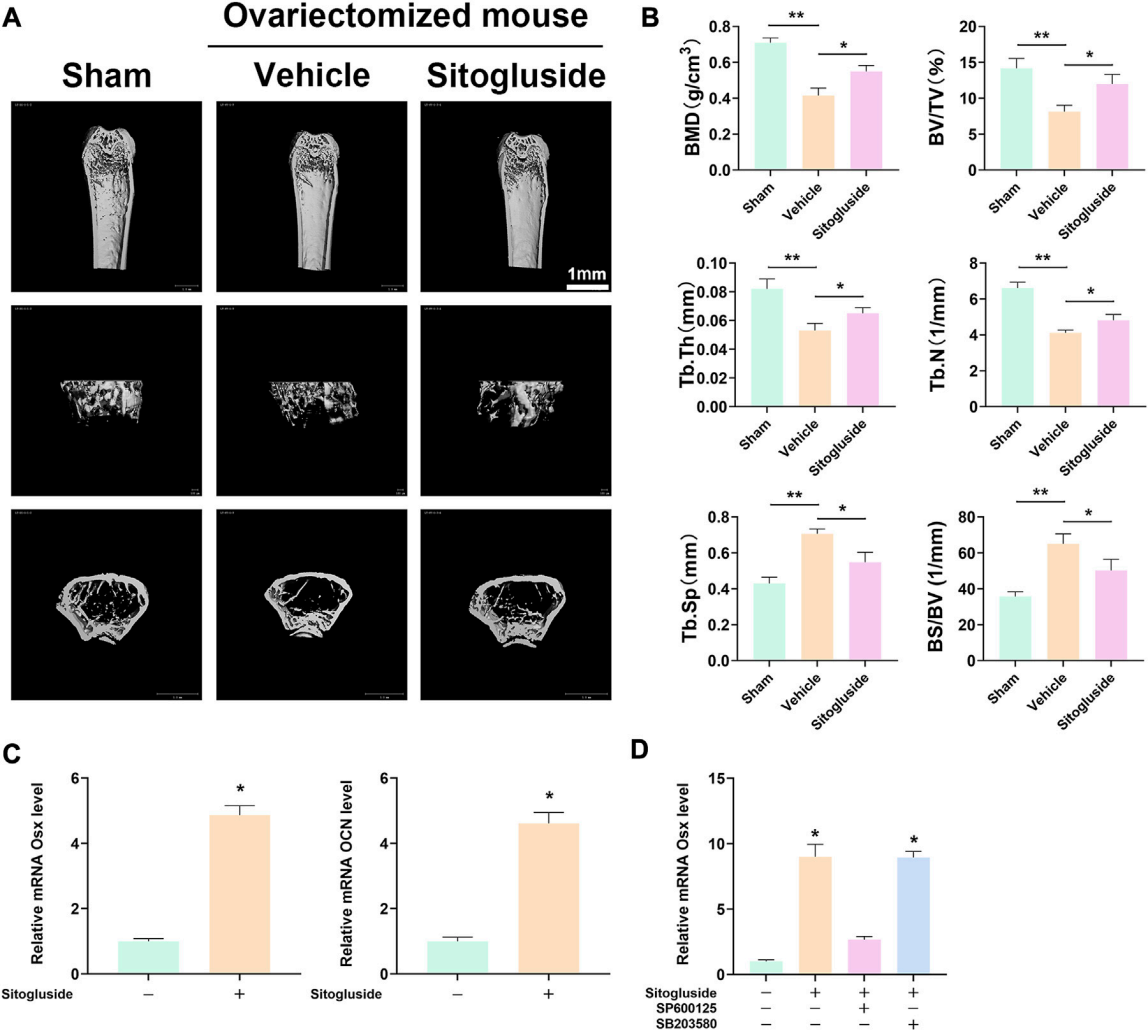
*In vivo* OVX model and q-PCR experiments to validate the potential anti-OP effect of sitogluside. **(A)** μCT scanning of mice tibia showing the bone loss alleviated by sitogluside’s efficiency. **(B)** Statistical results of osteoporosis phenotype parameters of OVX mice, including BMD, Tb. Th, BV/TV, and Tb. N. **(C)** q-PCR results of osteogenic biomarker, which is shown to promote the osteogenic effect of sitogluside. **(D)** q-PCR results of OSX, which, treated with sitogluside or combined with the specific inhibitor of JNK (SP600125) and p38 (SB203580), indicates that sitogluside could target the JNK pathway to promote osteogenic genes in the treatment of OP.

The OVX model was employed to further investigate the anti-osteoporotic effects of sitogluside *in vivo*. As depicted in Figure 7A, the μCT scan results demonstrated a significant loss of bone in the vehicle group (OVX mice) compared to the sham group, confirming the successful establishment of the OVX model. Additionally, the sitogluside-treated group exhibited a mitigating effect on bone loss compared to the vehicle group. Specifically, the sitogluside-treated OVX mice demonstrated increased bone mineral density (BMD), trabecular thickness (Tb. Th), bone volume fraction (BV/TV), and trabecular number (Tb. N), while exhibiting a decreased bone-surface-to-bone-volume ratio (BS/BV) and trabecular separation (Tb. Sp). These findings collectively indicate the anti-osteoporotic efficacy of sitogluside in the OVX mouse model (Figure 7B). Furthermore, histological examination of major organs in the drug-treated group, including the heart, liver, spleen, lungs, and kidneys, was conducted using (H&E) staining. Additionally, mouse serum was analyzed to assess cardiac, hepatic, and renal functions. As depicted in Supplementary Figure S1, the experimental results indicate that sitogluside did not exert significant toxic effects on mouse organs.

### 3.9 Effects of sitogluside on osteoblast-related genes

To gain further insights into the underlying mechanism of sitogluside’s anti-osteoporotic effects, we examined the expression of osteogenic-related genes using q-PCR and Western blot. As shown in Figure 7, treatment with sitogluside for 48 h resulted in a significant increase in the expression of osteogenic markers, such as Osterix (OSX) and osteocalcin (OCN). These findings suggest that sitogluside has the potential to promote osteogenic activity. Additionally, we investigated the core target and pathway associated with sitogluside in the treatment of osteoporosis via blocking the JNK and p38 cascade (Figure 7D). Our results revealed that targeting JNK could reduce the osteogenic efficacy of sitogluside while blocking the p38 pathway without significant expression changes of OSX. To further investigate whether sitogluside exerts its osteogenic effects through the JNK pathway, we employed shRNA to silence the JNK pathway. As shown in Supplementary Figure S3C, compared to the sitogluside group, silencing the JNK pathway with shRNA (shGnai3) resulted in a partial reduction in the expression levels of osteogenic markers, including Runx2, OSX, OCN, and ALP. However, when treated with the p-JNK activator anisomycin (ASM), these osteogenic markers increase. Moreover, as shown in Supplementary Figures S3A, B, the phosphorylation level of p-JNK increases with sitogluside intervention. These further suggest that sitogluside may promote osteoblast differentiation through the JNK signaling pathway. We also investigated the effect of sitogluside on the Smad 1/5/8 protein in the BMP signaling pathway, which also showed increasing expression (Supplementary Figures S3A, B).

## 4 Discussion

Osteoporosis is regarded as a silent disease without clinical symptoms before complications are apparent (Anthamatten and Parish, 2019). Current conventional therapy mainly focuses on symptom prevention with long-term supplements of calcium and on intervention to regulate bone metabolism. The curative effects depend on the individual response and have mild or severe side effects (Ensrud and Crandall, 2017). AB and DA have been two of the most important herbs for bone diseases in TCM therapy for more than 2,000 years (He et al., 2017; Tao et al., 2020), and play an important role in many classic anti-osteoporosis drugs. Recently, researchers have isolated more than 100 ingredients and verified numerous bioactivities (He et al., 2017; Tao et al., 2020). Our research obtained 31 bioactive compounds of AB–DA based on the screening criteria and applied the main therapeutic compounds to anti-osteoporosis. Compounds of AB–DA such as baicalin, kaempferol, oleanol, quercetin, and sitosterol are widespread in herbal medicine, and many studies have shown their anti-osteoporosis activity. It has been reported that kaempferol could ameliorate the inhibitory effects of osteogenesis by activating JNK and p38 pathways in the glucocorticoid-induced and ovariectomy-induced osteoporosis model (Wong et al., 2019). Vakili et al. (2021) also constructed an OVX osteoporosis model treated with quercetin; results showed that it might modulate cell autophagy and apoptosis to alleviate osteoporosis; the potential mechanisms mainly involve Wnt, NF-κB, and MAPK cascades. Moreover, asperosaponin VI is the quality indicator of DA and could induce the differentiation of osteoblastic cells by increased expression of BMP2, promote osteogenesis and angiogenesis via regulating the OPG/RANKL signaling pathway, and inhibit the differentiation of osteoclast (Chen et al., 2022). β-Ecdysterone is an iconic ingredient of AB and could upregulate the activity of alkaline phosphatase in mesenchymal stem cells by modulating the expression of estrogen receptors (Gao et al., 2008) and could also inhibit apoptosis and autophagy induced by the dexamethasone of osteoblast cells *in vivo* and *in vitro* (Tang et al., 2018). This body of evidence suggests that the numerous bioactive compounds retrieved from AB–DA have certain anti-OP effects and partly confirm our findings; however, more accurate and in-depth study is still necessary to explore the AB–DA herb pair.

After screening the core therapeutic ingredients, a PPI network was constructed to determine core targets; our results showed that AKT1, CASP3, EGFR, ESR1, IGF1, MAPK1, and MAPK14 are important for AB–DA treatment of osteoporosis. It is widely reported that RAC-alpha serine/threonine-protein kinase (AKT1) regulates a series of biological processes, including cell proliferation, growth, metabolism, and angiogenesis (Heron-Milhavet et al., 2011). A targeted knockdown AKT1 mouse model showed that AKT1 deficiency would induce osteoclast-osteogenesis disorder and diminish the RANKL (NF-κB ligand) and MCSF (macrophage colony-stimulating factor) receptors on multinucleated osteoclasts. This is evidence for AKT1 as an intermediator to regulate osteoblast and osteoclast differentiation (Mukherjee and Rotwein, 2012). Wang et al. (2022) also showed that modulating the expression of AKT1 could relieve osteoporosis. Epidermal growth factor receptor (EGFR) could activate the downstream of MEK-ERK, PI3K-AKT, and NF-κB signaling, transferring extracellular cues into cellular response (Runkle et al., 2016). It was reported that promoting the phosphorylation of EGFR and ERK1/2 could alleviate the apoptosis induced by H_2_O_2_ of MC3T3-E1 (osteoblast cell) (Yang et al., 2019), exhibiting the potential targeted therapeutic value of EGFR on osteoporosis. Insulin-like growth factor I (IGF1) is the most abundant growth promotor in the bone matrix and also regulates glycogen synthesis in osteoblasts; it plays an important role in bone homeostasis maintenance and osteoblast differentiation by mediating the mTOR (mammalian target of rapamycin) signaling pathway (Xian et al., 2012). The results of molecular docking and molecular dynamics simulation also showed a stronger binding affinity between AB–DA compounds and IGF1. All the aforementioned evidence shows that the hub genes regulated by AB–DA compounds are important and meaningful in the treatment of osteoporosis.

The GO/KEGG pathway enrichment analysis of 83 potential therapeutic targets showed that AB–DA anti-osteoporosis mainly involved the MAPK signaling pathway, osteoclast differentiation, and the PI3K-Akt signaling pathway. As shown in Figure 4E, the colored target details of the MAPK pathway—ERK (MAPK1), JNK(MAPK8), and p38 (MAPK14)—are three classical cascades of the MAPK pathway, all of which were involved in the potential mechanism of AB–DA treating osteoporosis. ERK cascade mediated cell growth and differentiation via cytoskeletal rearrangement, and upregulating the phosphorylation of ERK (p-ERK) might promote osteoblast differentiation (Jing et al., 2019). The JNK and p38 cascades would be activated by extracellular stimulation, such as pro-inflammatory and physical stress. Lee et al. (2019) showed that downregulated osteoclast-related gene expression was associated with JNK cascade inhibition and that suppressing the p38 cascade would also relieve osteoporosis (Wang et al., 2018). Liu et al. (2022) indicated that vitexin could act against osteoporosis by promoting osteogenesis and angiogenesis in an ovariectomized rat model; the potential underlying mechanism might upregulate the PI3K-AKT cascade. Regulated PI3K-AKT signaling could also mediate the biological function of osteoclast (Jiang et al., 2022). Thus, our research mined the herb databases and screened therapeutic compounds of AB–DA with appropriate pharmacokinetic properties. In summary, the 31 therapeutic compounds have different targets and regulate different signaling pathways with a synergistic effect against osteoporosis, showing the complex molecular mechanisms with “multi-compound,” “multi-target,” and “multi-pathway.”

Furthermore, *in vitro* and *in vivo* experimental validation were both employed to explore the anti-osteoporotic effects of sitogluside (also known as daucosterol) and its underlying molecular mechanism. Some research has indicated the regulation of both the osteoblast and osteoclast formation of AB–DA (He et al., 2017; Tao et al., 2020). In this present research, our findings show the potential effects of sitogluside with osteoblast differentiation and mineralization, but the effects on osteoclast were not significant; this was also verified by the collaborative therapeutic effects of AB–DA compounds. Moreover, q-PCR and Western blot analyses showed that sitogluside might upregulate the JNK cascade to promote osteogenics, such as Runx2, Osx, and OCN, and knockdown or block with the inhibitor could partly reduce its efficacy. Previous research has also reported that daucosterol could increase the p-JNK expression to exert an anti-prostate cancer effect (Gao et al., 2019). Huang et al. reported that the JNK kinase pathway with downstream OSX belonged to the non-canonical Smad-independent BMP signaling pathway to promote osteogenics. Hence, our study illustrated that a potential mechanism for sitogluside in the treatment of osteoporosis was to promote the JNK pathway and non-canonical BMP signaling to regulate downstream osteogenic genes. In addition, the OVX mouse model also showed anti-osteoporosis efficacy *in vivo* without observable toxicity. Our results therefore highlight the potential therapeutic value of sitogluside in the treatment of osteoporosis. However, further systematic and in-depth research is required to explore other bioactive compounds of the AB–DA combination.

There were some limitations to our research. First, it is based on the bioinformatic analysis of network pharmacology, molecular docking, and molecular dynamics simulation. The results illustrated the core therapeutic ingredients, core targets, and core signaling pathways of AB–DA in the treatment of osteoporosis, although we have preliminarily verified the anti-osteoporotic effect of sitogluside. Our findings thus need more wet experiments *in vitro* and *in vivo* for corroboration. The second limitation is that all the bioactive compounds were filtered by the ADME properties; the content of specific ingredients were not considered, and the toxicological information was ignored. Therefore, the next task for us is to comprehensively verify our network pharmacological findings with experiments and evaluate the biosafety of these ingredients for optimally utilizing AB–DA treatment of osteoporosis during clinical practice (Zou et al., 2021).

## 5 Conclusion

This study represents the first comprehensive investigation into the bioactive compounds of the AB–DA herb pair. Our findings illustrate that the primary therapeutic compounds responsible for treating osteoporosis are iridoids, steroids, and flavonoids. Additionally, we propose that the underlying mechanisms of action may involve targeting key core targets, including MAPK1, MAPK8, and MAPK14, to modulate the MAPK cascade (ERK/JNK/p38). Furthermore, *in vitro* and *in vivo* experiments have provided preliminary validation of the anti-osteoporotic effect of the most potent bioactive compound, sitogluside. Nevertheless, further in-depth experiments are needed to fully harness the therapeutic potential of AB–DA for treating osteoporosis in clinical practice.

## Data Availability

The datasets presented in this study can be found in online repositories. The names of the repository/repositories and accession number(s) can be found in the article/Supplementary Material.

## Supplementary material

SUPPLEMENTARY TABLE S1 Detailed information of AB–DA herb pair bioactive compounds.

SUPPLEMENTARY TABLE S2 Protein name and Entrez ID of all targets.
