# Supplementary figures and images for: Unraveling the potential mechanisms of the anti-osteoporotic effects of the Achyranthes bidentata–Dipsacus asper herb pair: a network pharmacology and experimental study
Source: Front Pharmacol. 2023 Oct 2;14:1242194. doi: 10.3389/fphar.2023.1242194 (PMC10577322; doi:10.3389/fphar.2023.1242194)

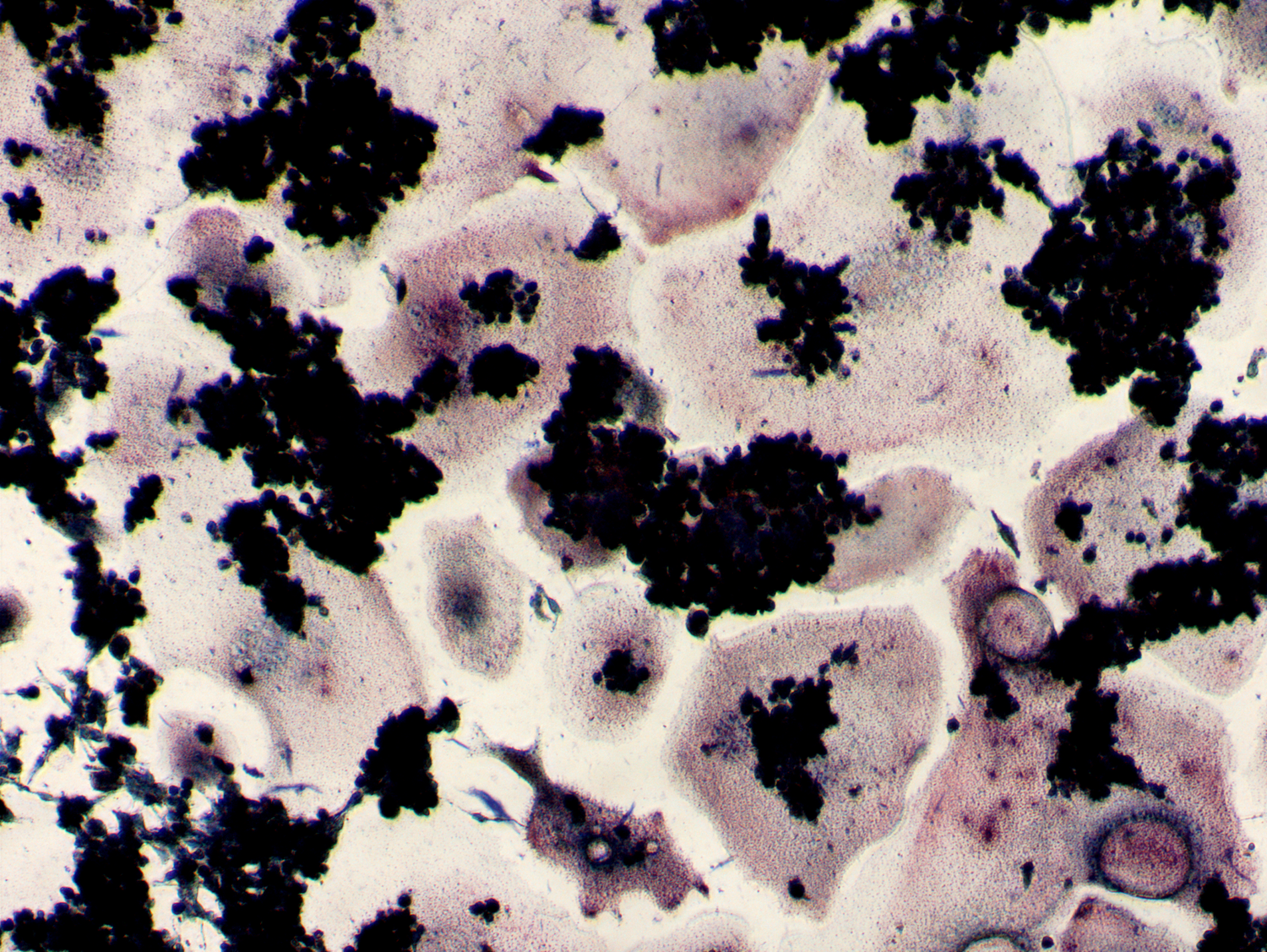

Supplement: Supplementary file 3 [file DataSheet4.ZIP › TRAP staining(osteoclast)/0.4μM.tif]

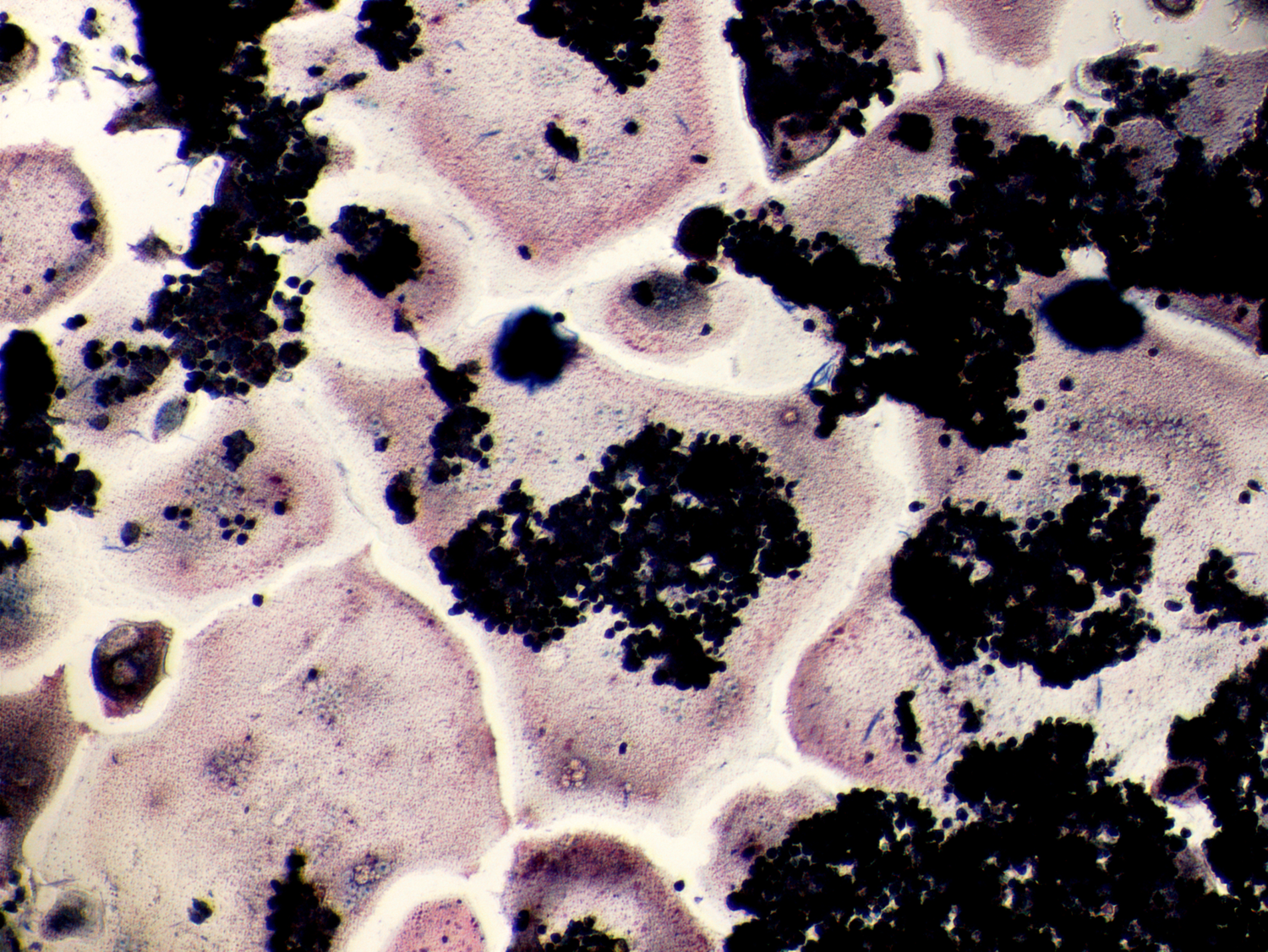

Supplement: Supplementary file 3 [file DataSheet4.ZIP › TRAP staining(osteoclast)/40μM.tif]

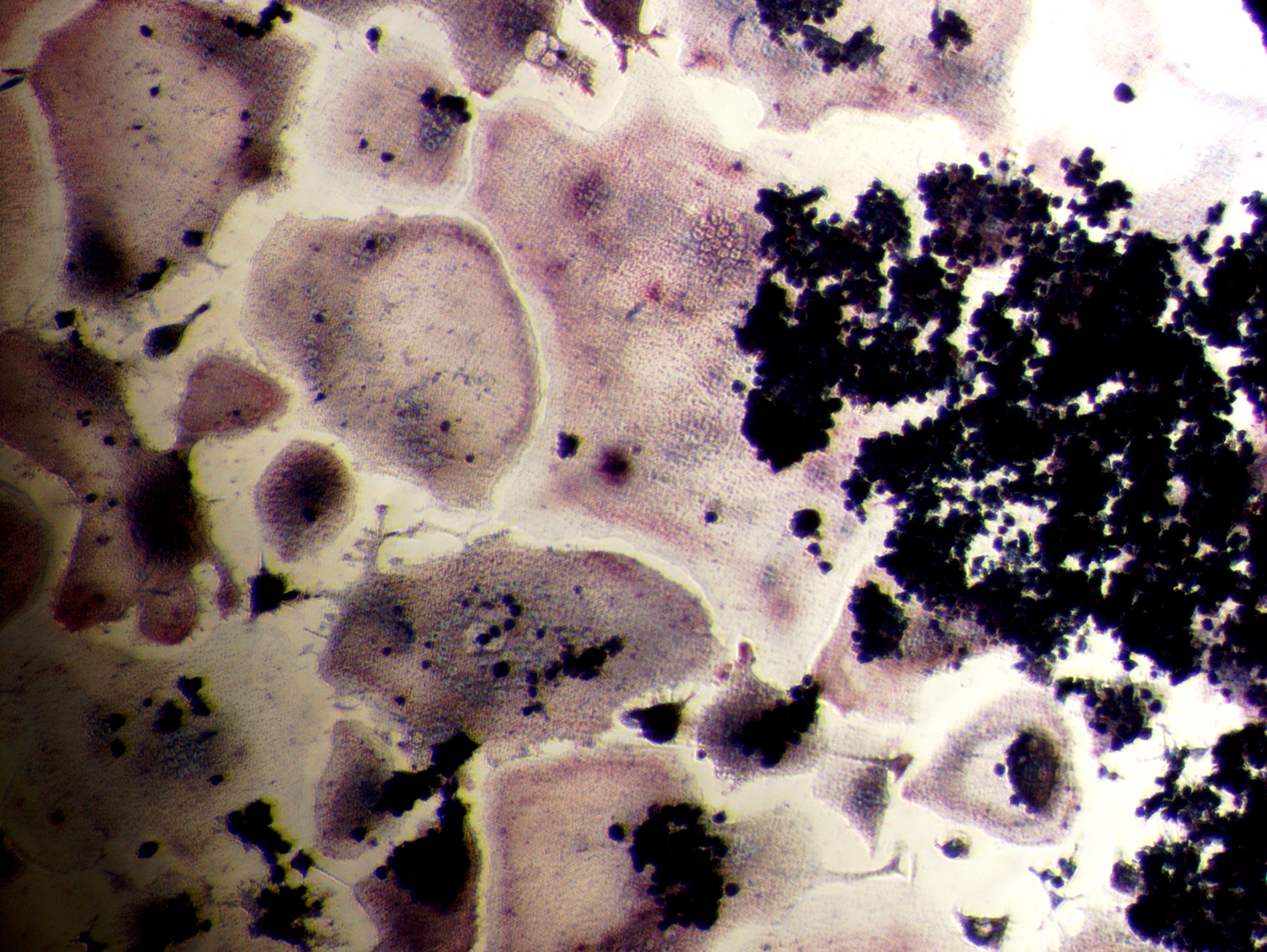

Supplement: Supplementary file 3 [file DataSheet4.ZIP › TRAP staining(osteoclast)/4μM.tif]

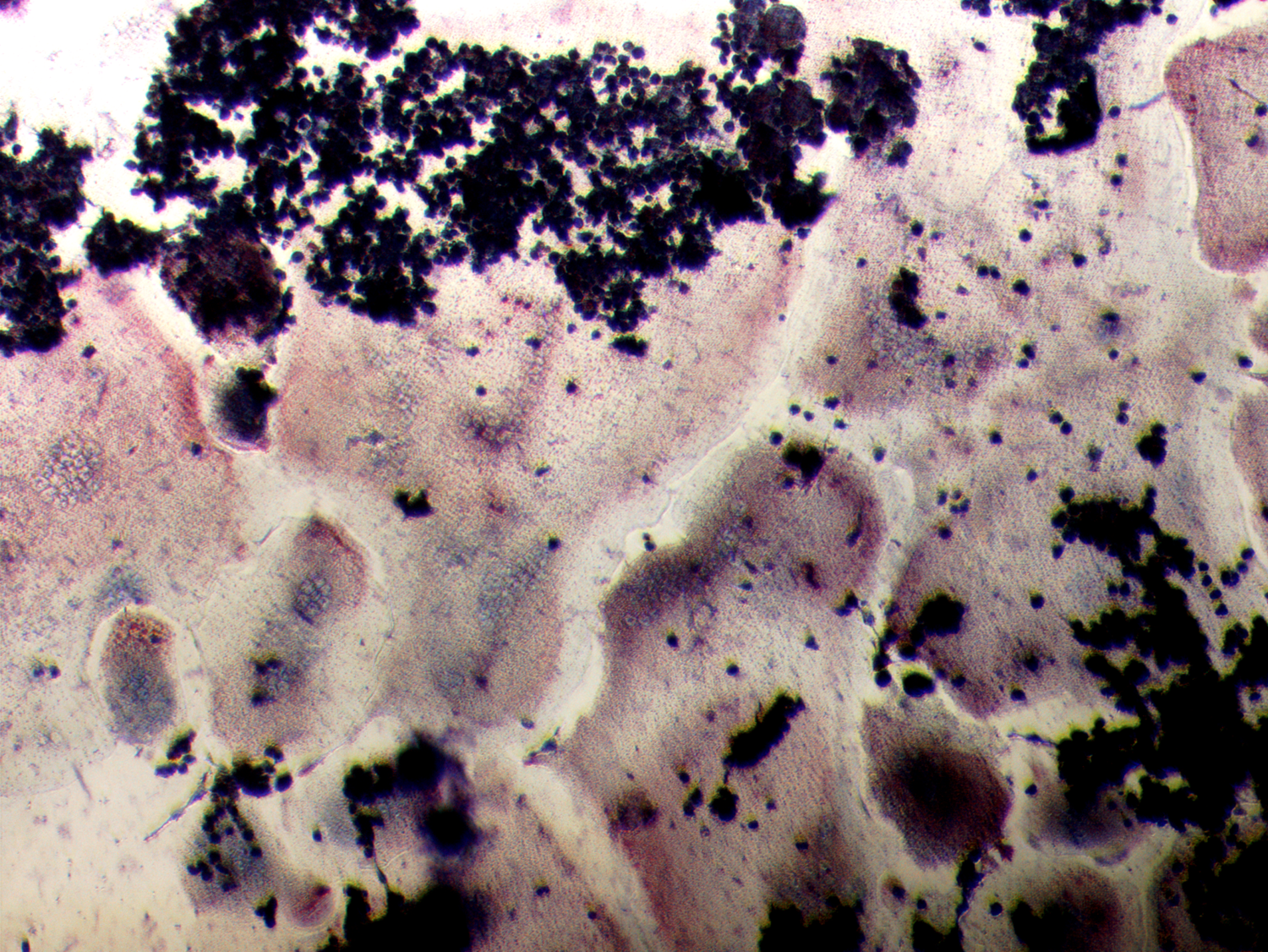

Supplement: Supplementary file 3 [file DataSheet4.ZIP › TRAP staining(osteoclast)/Ctrl.tif]

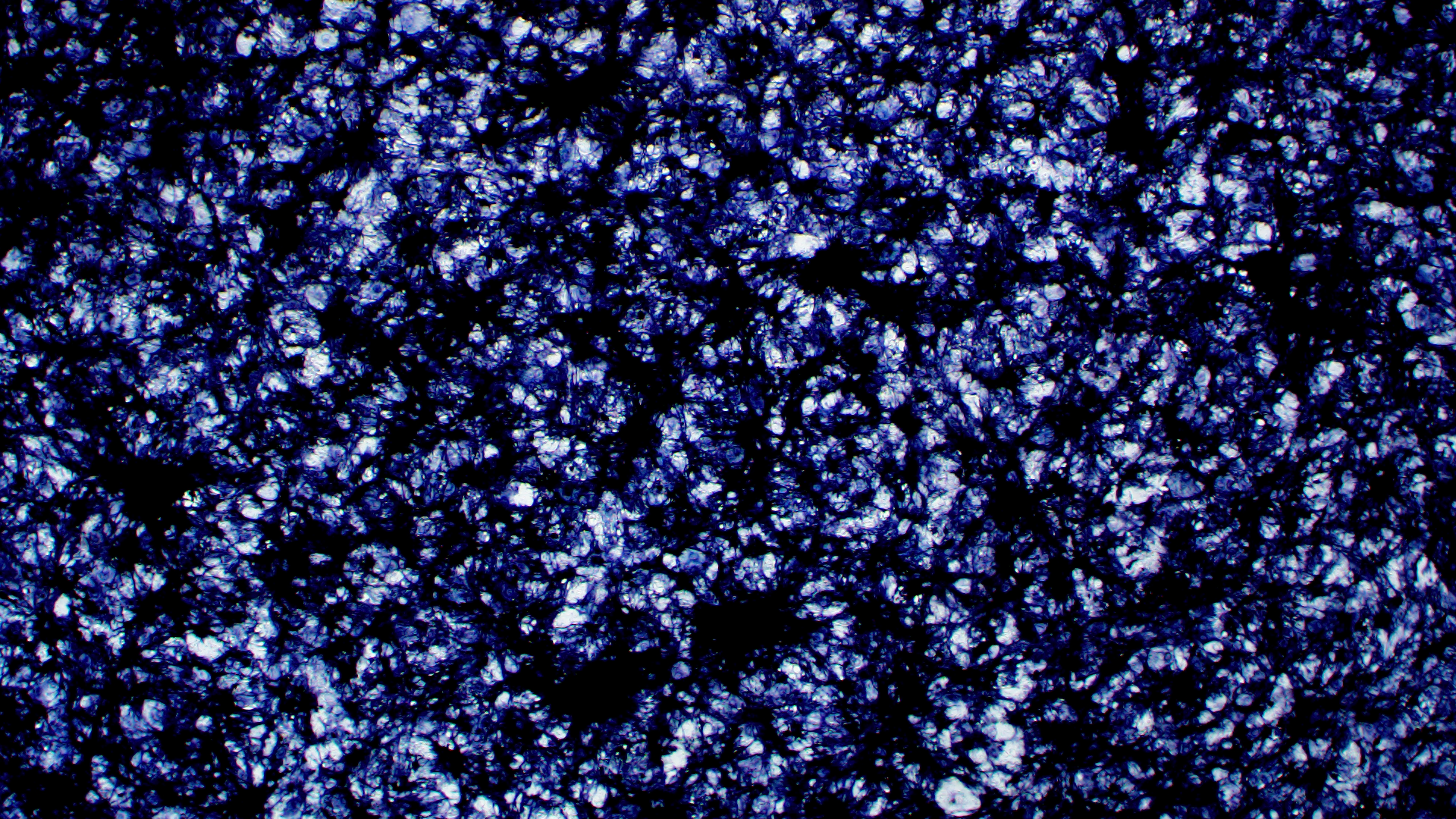

Supplement: Supplementary file 4 [file DataSheet1.zip › ALP staining/Sit+shGnai3+ASM.tif]

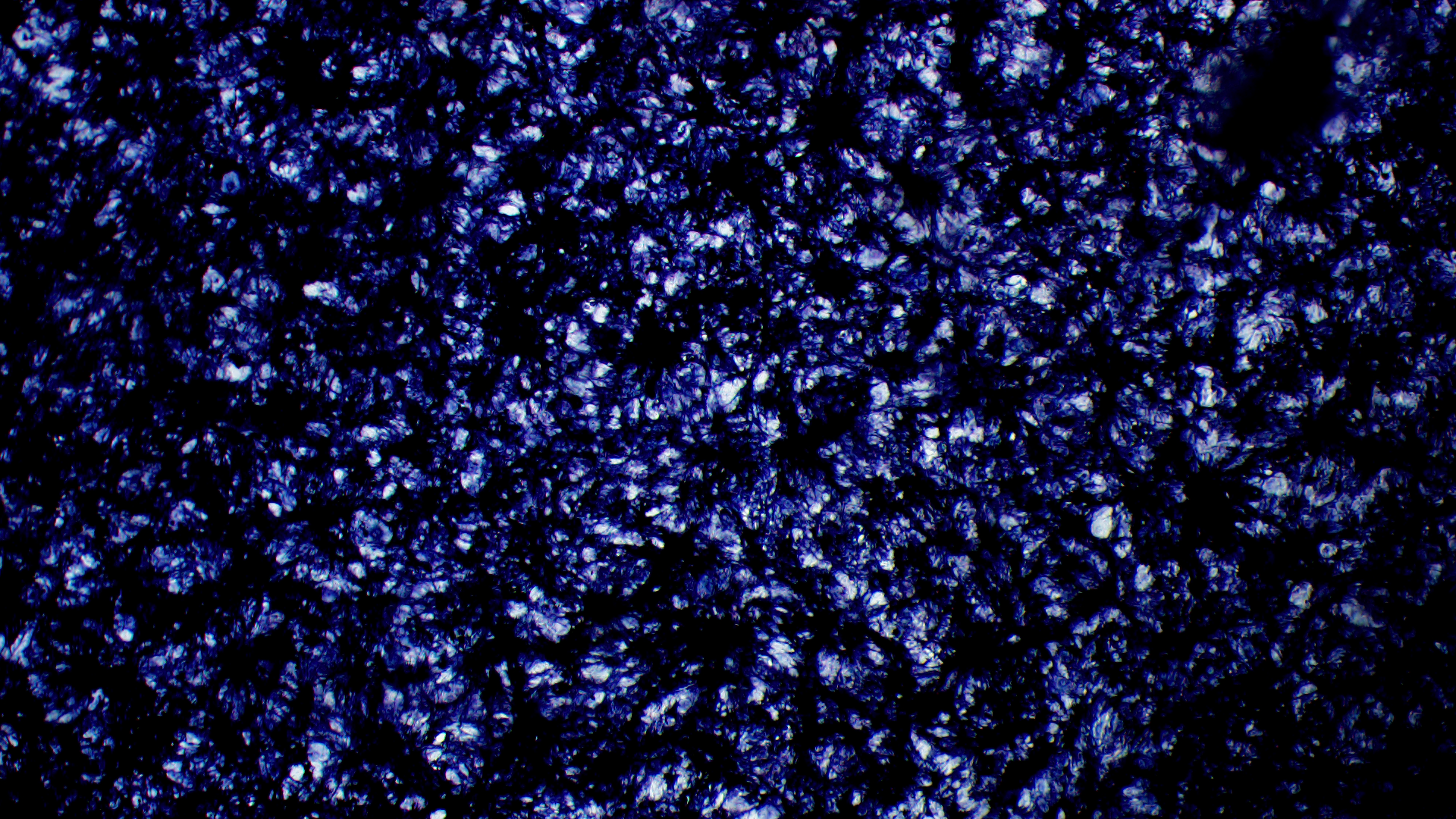

Supplement: Supplementary file 4 [file DataSheet1.zip › ALP staining/Sit.tif]

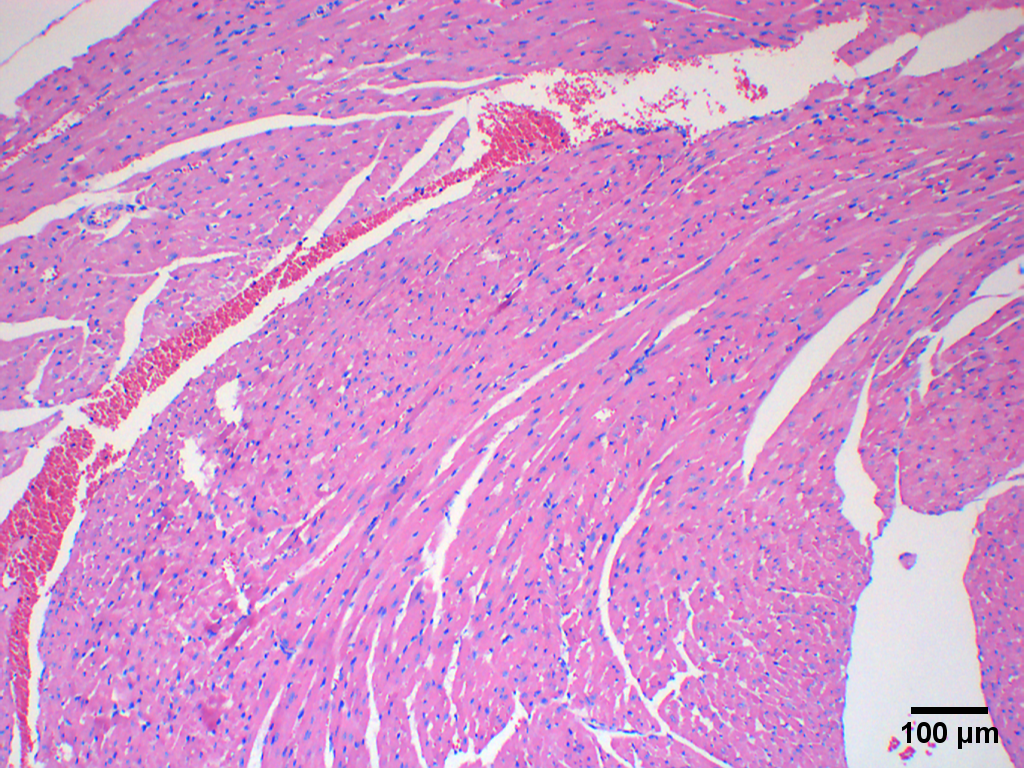

Supplement: Supplementary file 5 [file DataSheet6.ZIP › HE staining(heart, liver, spleen, lung, and kidney)/Heart-100.tif]

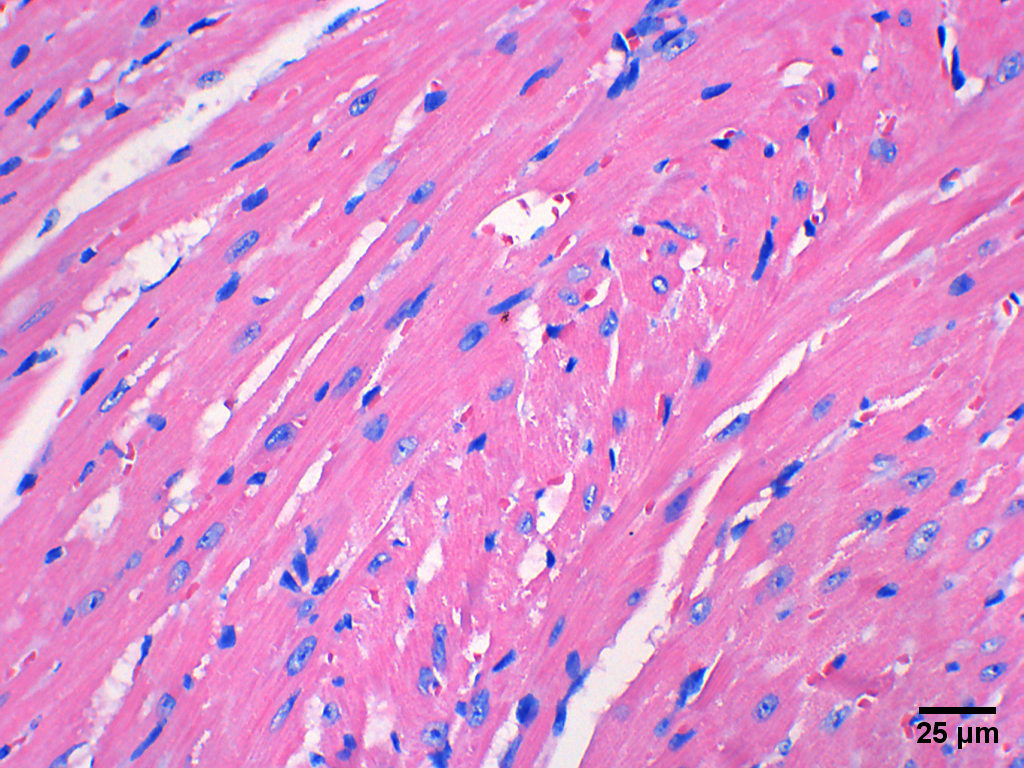

Supplement: Supplementary file 5 [file DataSheet6.ZIP › HE staining(heart, liver, spleen, lung, and kidney)/Heart-400.tif]

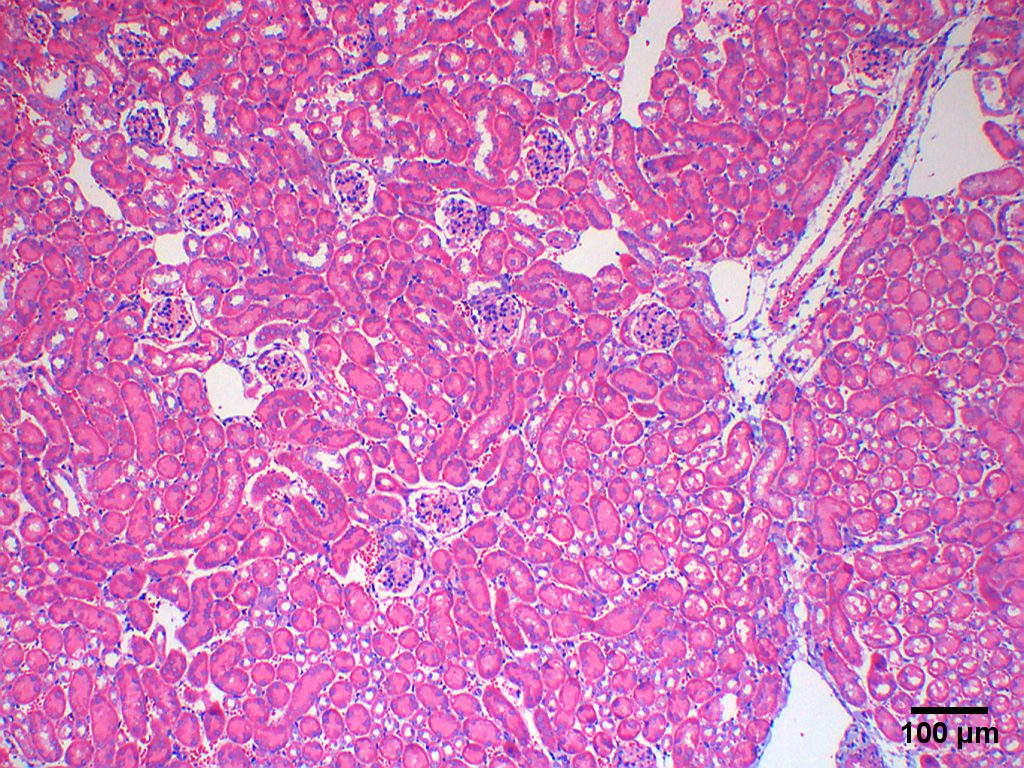

Supplement: Supplementary file 5 [file DataSheet6.ZIP › HE staining(heart, liver, spleen, lung, and kidney)/Kidney-100.tif]

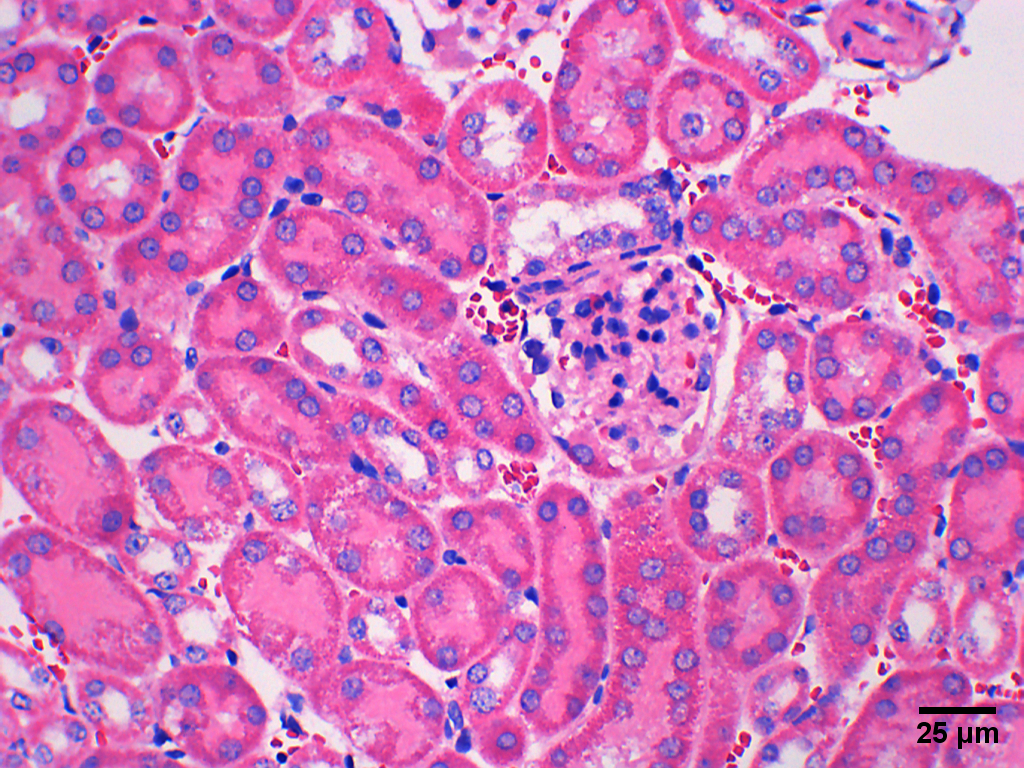

Supplement: Supplementary file 5 [file DataSheet6.ZIP › HE staining(heart, liver, spleen, lung, and kidney)/Kidney-400.tif]

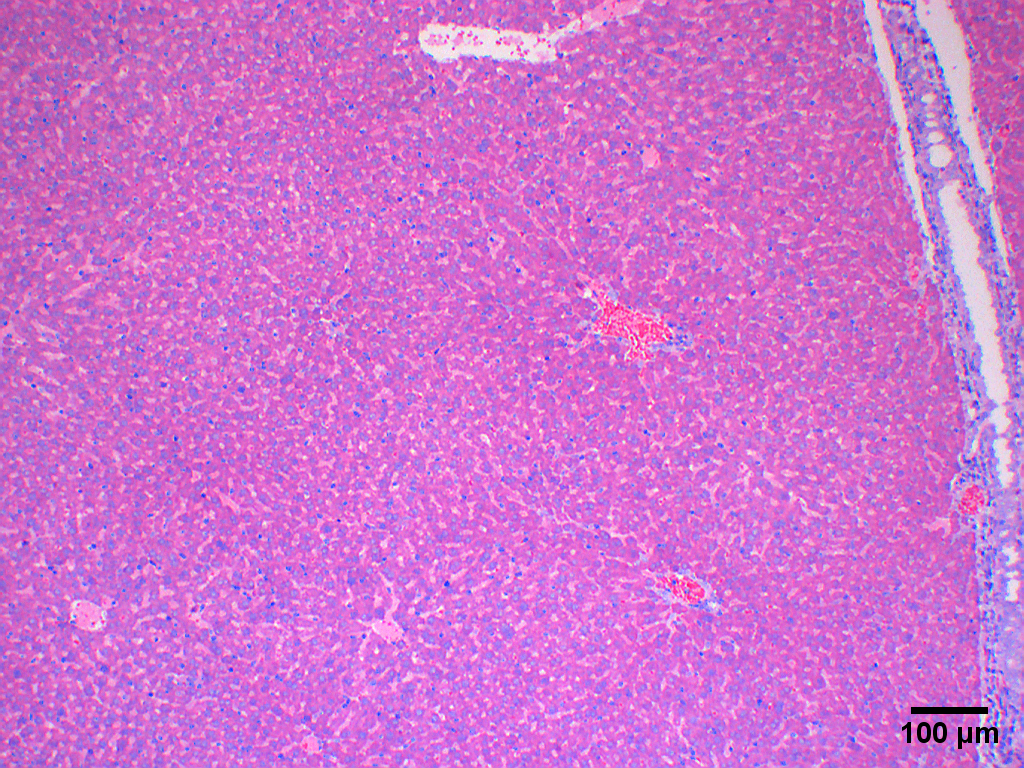

Supplement: Supplementary file 5 [file DataSheet6.ZIP › HE staining(heart, liver, spleen, lung, and kidney)/Liver-100.tif]

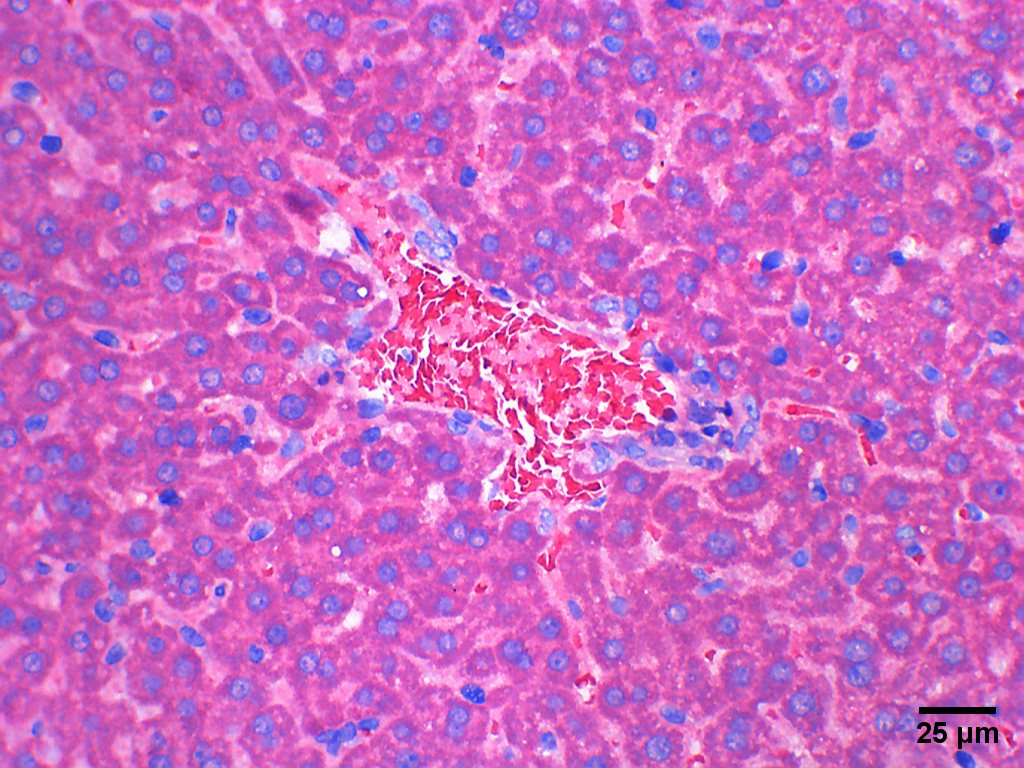

Supplement: Supplementary file 5 [file DataSheet6.ZIP › HE staining(heart, liver, spleen, lung, and kidney)/Liver-400.tif]

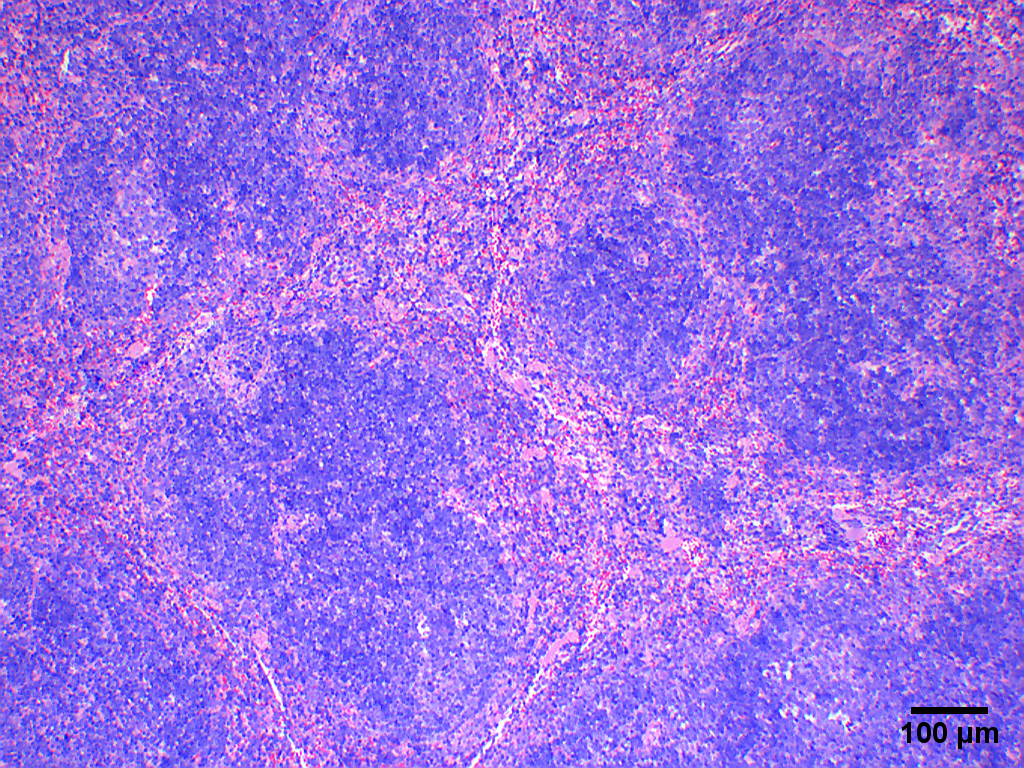

Supplement: Supplementary file 5 [file DataSheet6.ZIP › HE staining(heart, liver, spleen, lung, and kidney)/Spleen-100.tif]

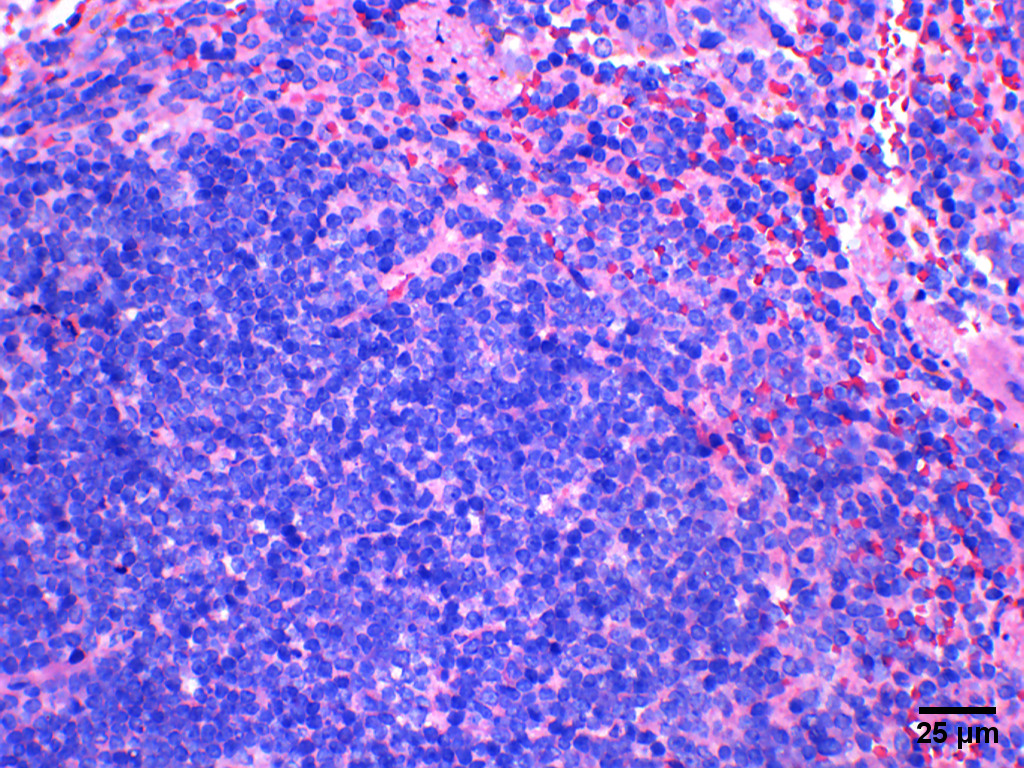

Supplement: Supplementary file 5 [file DataSheet6.ZIP › HE staining(heart, liver, spleen, lung, and kidney)/Spleen-400.tif]

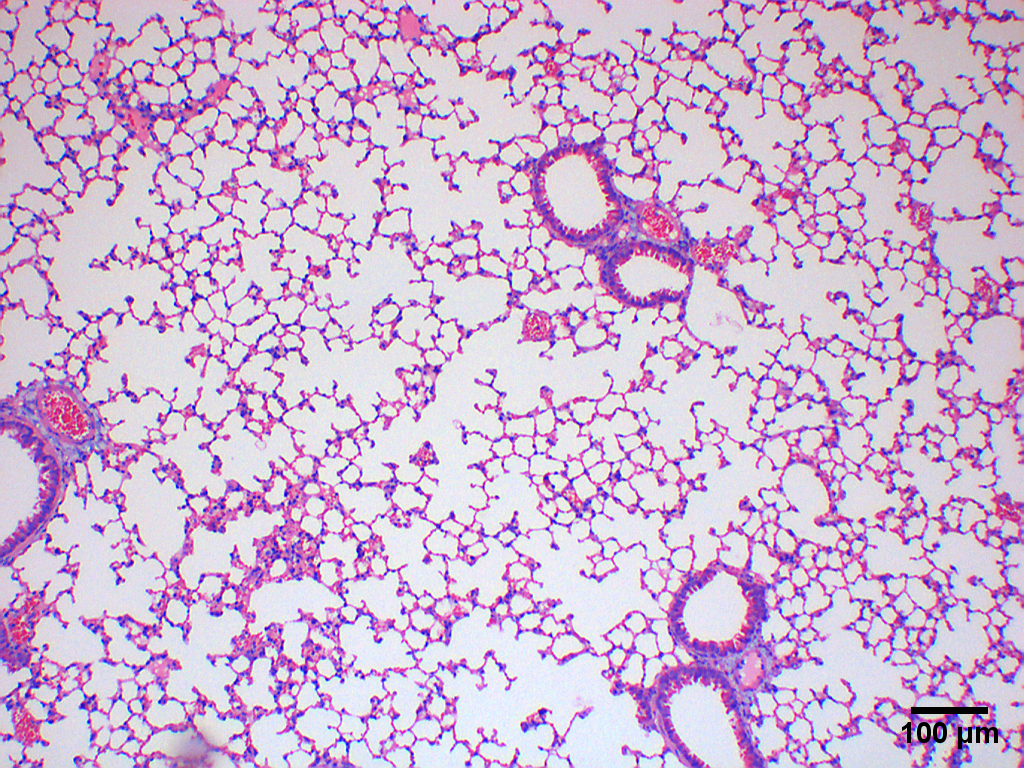

Supplement: Supplementary file 5 [file DataSheet6.ZIP › HE staining(heart, liver, spleen, lung, and kidney)/lung-100.tif]

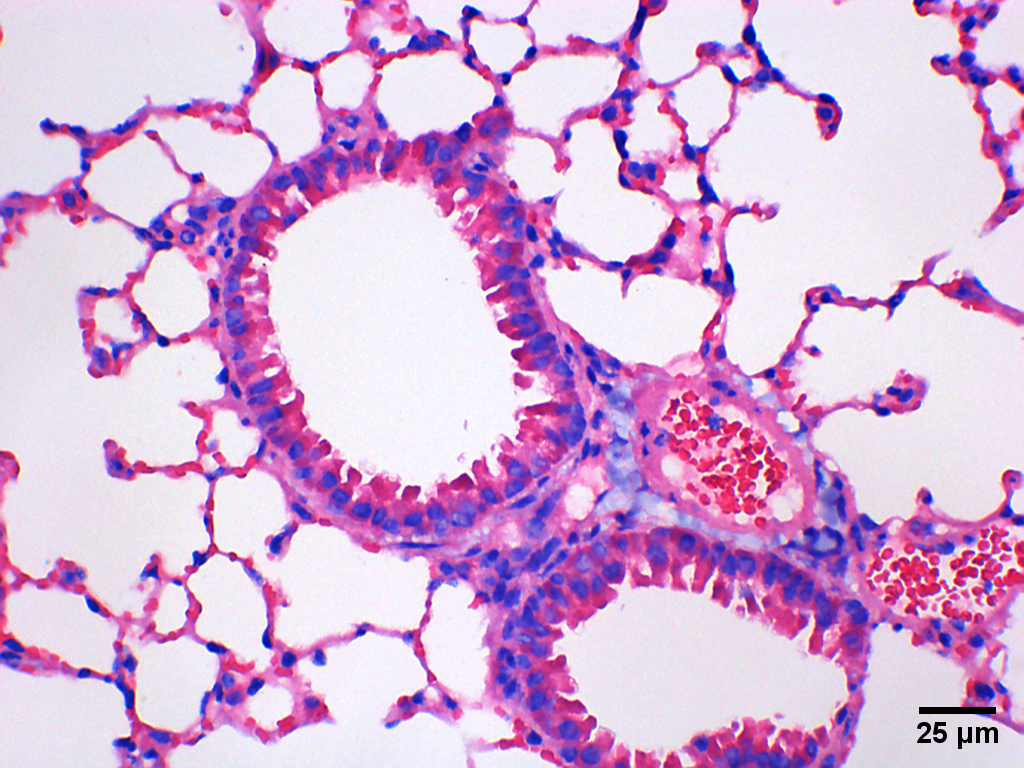

Supplement: Supplementary file 5 [file DataSheet6.ZIP › HE staining(heart, liver, spleen, lung, and kidney)/lung-400.tif]

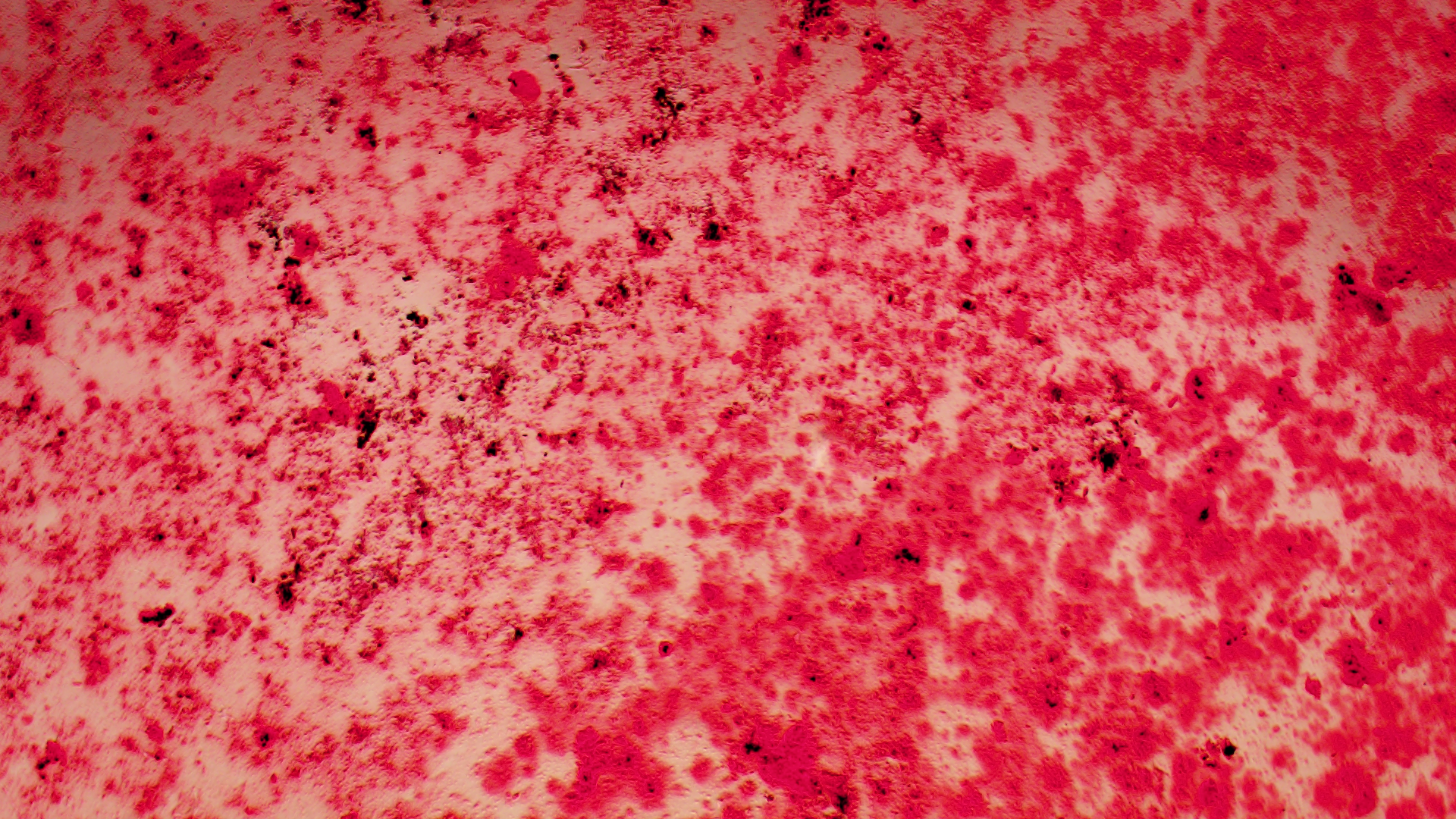

Supplement: Supplementary file 6 [file DataSheet2.zip › ARS staining/Sit+shGnai3+ASM.tif]

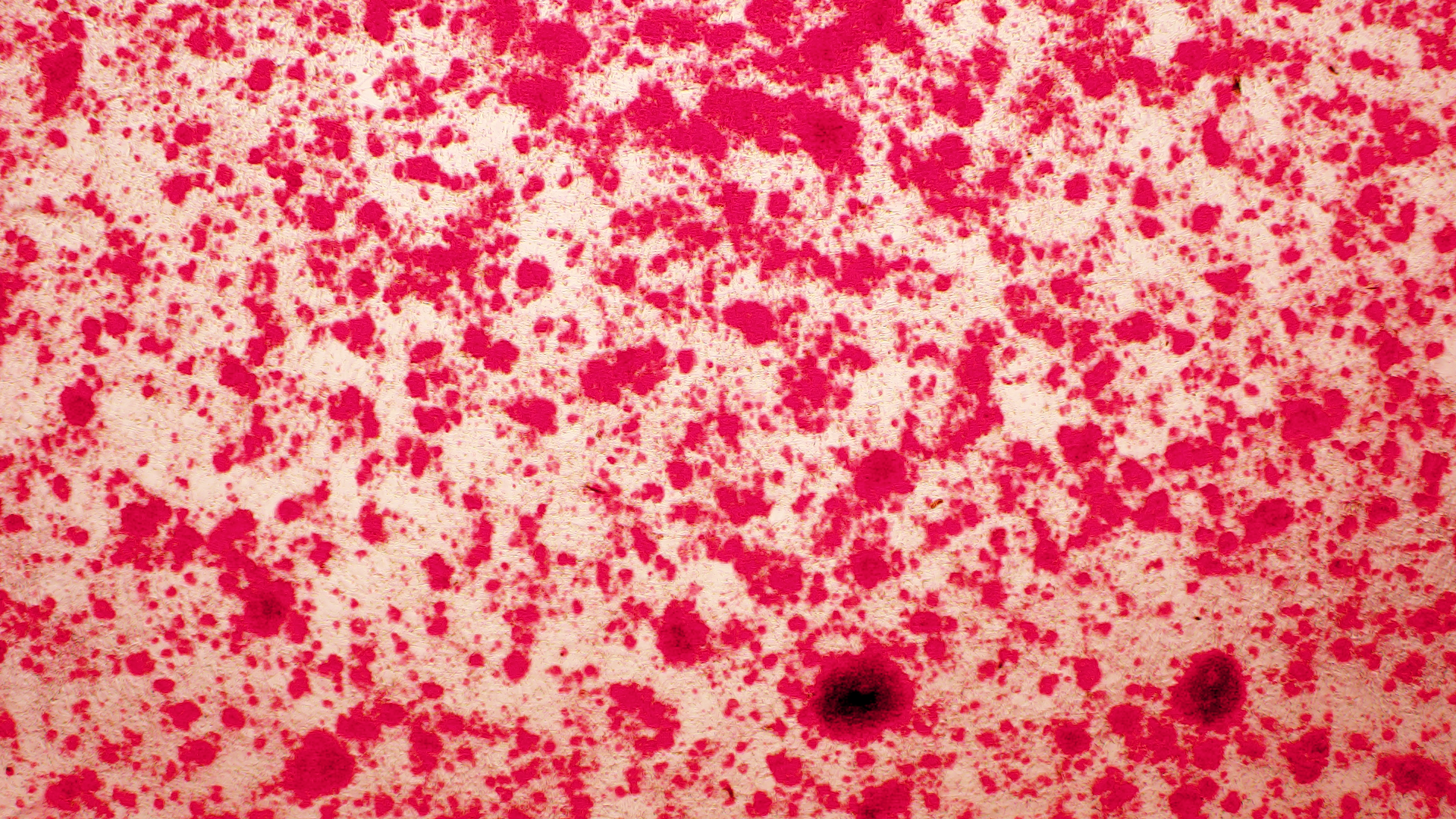

Supplement: Supplementary file 6 [file DataSheet2.zip › ARS staining/Sit+shGnai3.tif]

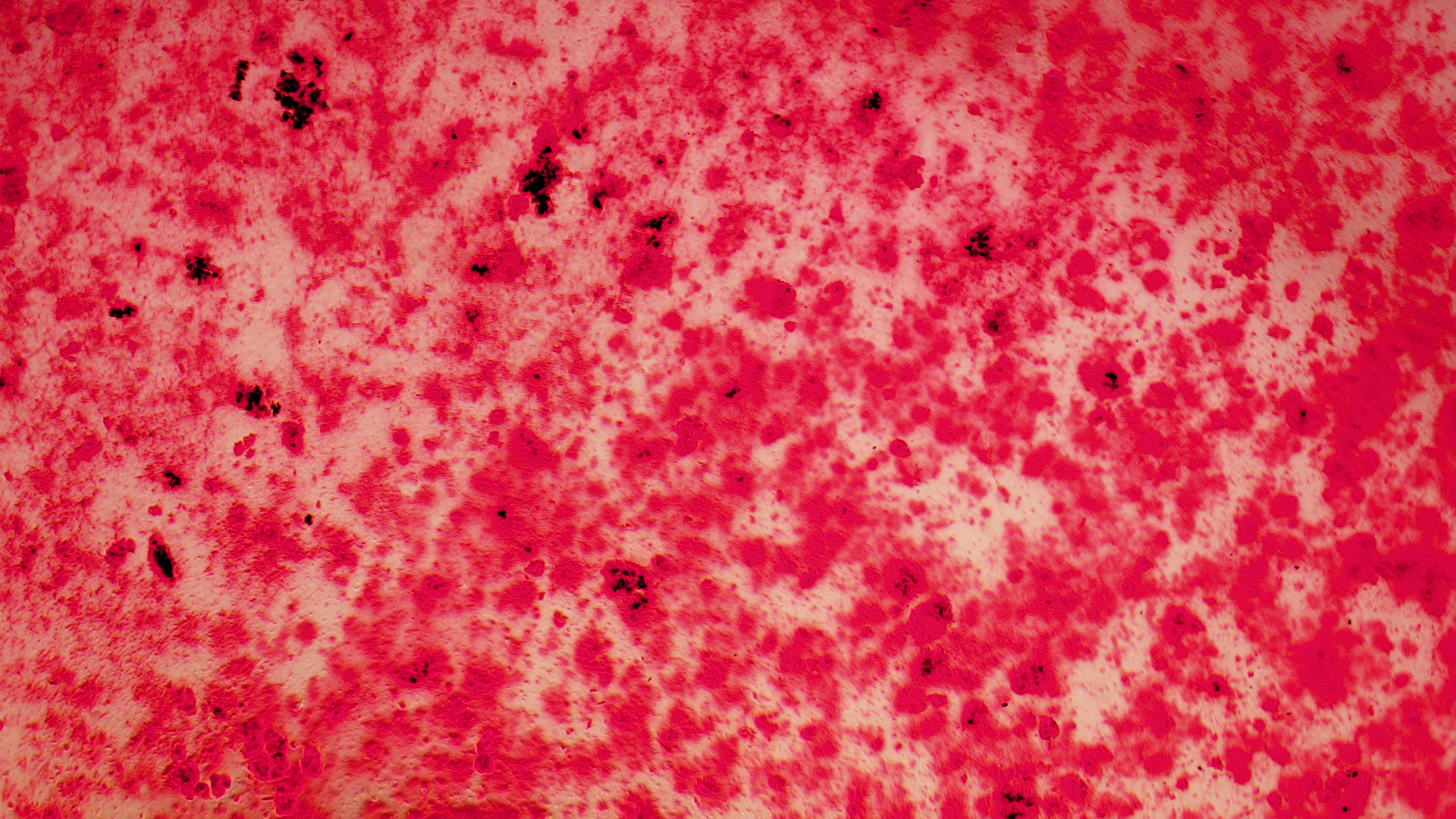

Supplement: Supplementary file 6 [file DataSheet2.zip › ARS staining/Sit.tif]

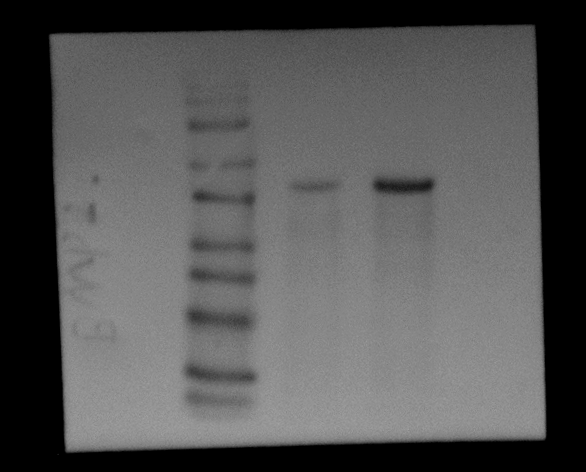

Supplement: Supplementary file 8 [file DataSheet5.ZIP › Western blotting/BMP2.tif]

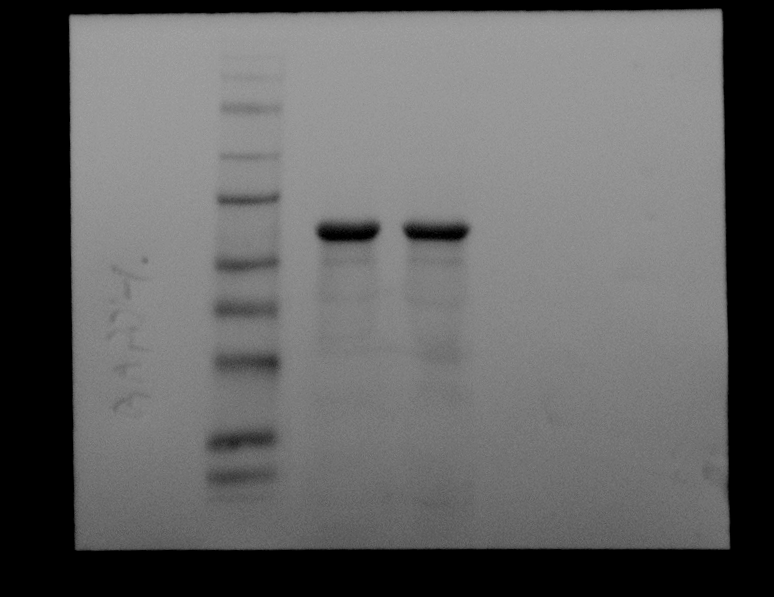

Supplement: Supplementary file 8 [file DataSheet5.ZIP › Western blotting/GAPDH.tif]

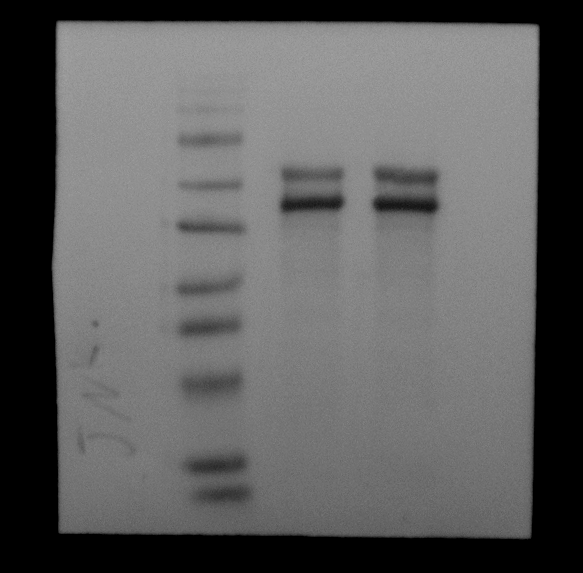

Supplement: Supplementary file 8 [file DataSheet5.ZIP › Western blotting/JNK.tif]

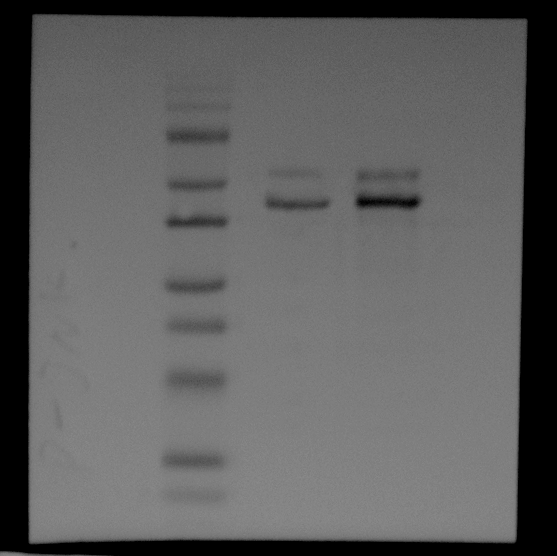

Supplement: Supplementary file 8 [file DataSheet5.ZIP › Western blotting/P-JNK.tif]

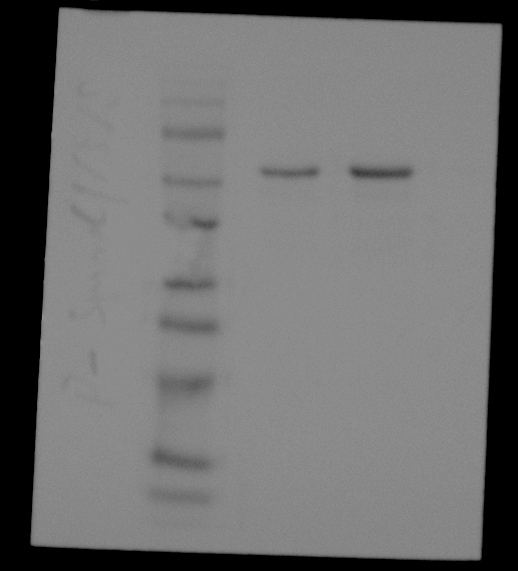

Supplement: Supplementary file 8 [file DataSheet5.ZIP › Western blotting/P-smad 1 5 8.tif]

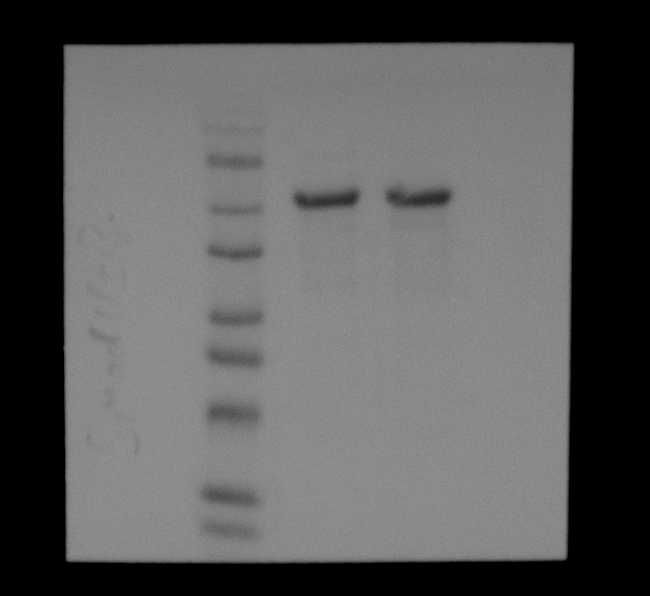

Supplement: Supplementary file 8 [file DataSheet5.ZIP › Western blotting/smad 1 5 8.tif]
