# Supplementary material for: Unraveling the potential mechanisms of the anti-osteoporotic effects of the Achyranthes bidentata–Dipsacus asper herb pair: a network pharmacology and experimental study
Source: Front Pharmacol. 2023 Oct 2;14:1242194. doi: 10.3389/fphar.2023.1242194 (PMC10577322; doi:10.3389/fphar.2023.1242194)
Supplement: Supplementary file 10 [file DataSheet1.docx]

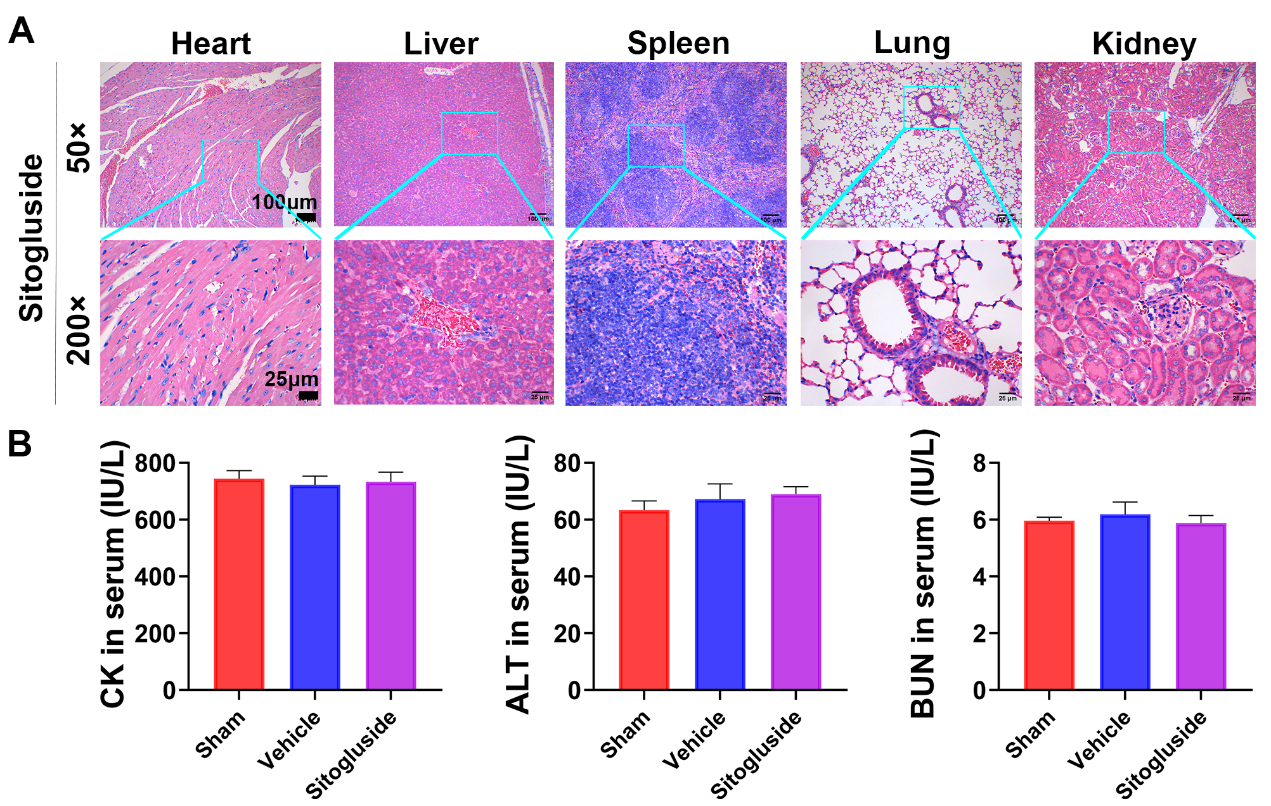


**Figure. S1. Safety evaluation of sitogluside *in vivo*.** **A.** Histological examination of the hearts, livers, spleens, lungs, and kidneys of mice in the sitogluside (Sit, 10mg/kg) group. **B.** Hematological parameters of mice. Creatine kinase (CK), alanine transaminase (ALT), and blood urea nitrogen (BUN).


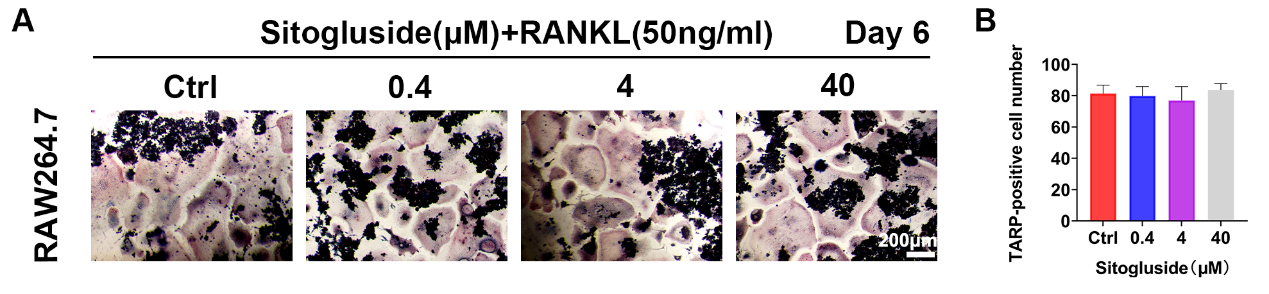


**Figure. S2. Effects of sitogluside on RANKL-mediated osteoclast differentiation *in vitro*.** **A.** Osteoclast differentiation of RAW 264.7 cells (preosteoclast) in vitro after RANKL and sitogluside administration. **B.** Quantification of osteoclast formation.

**
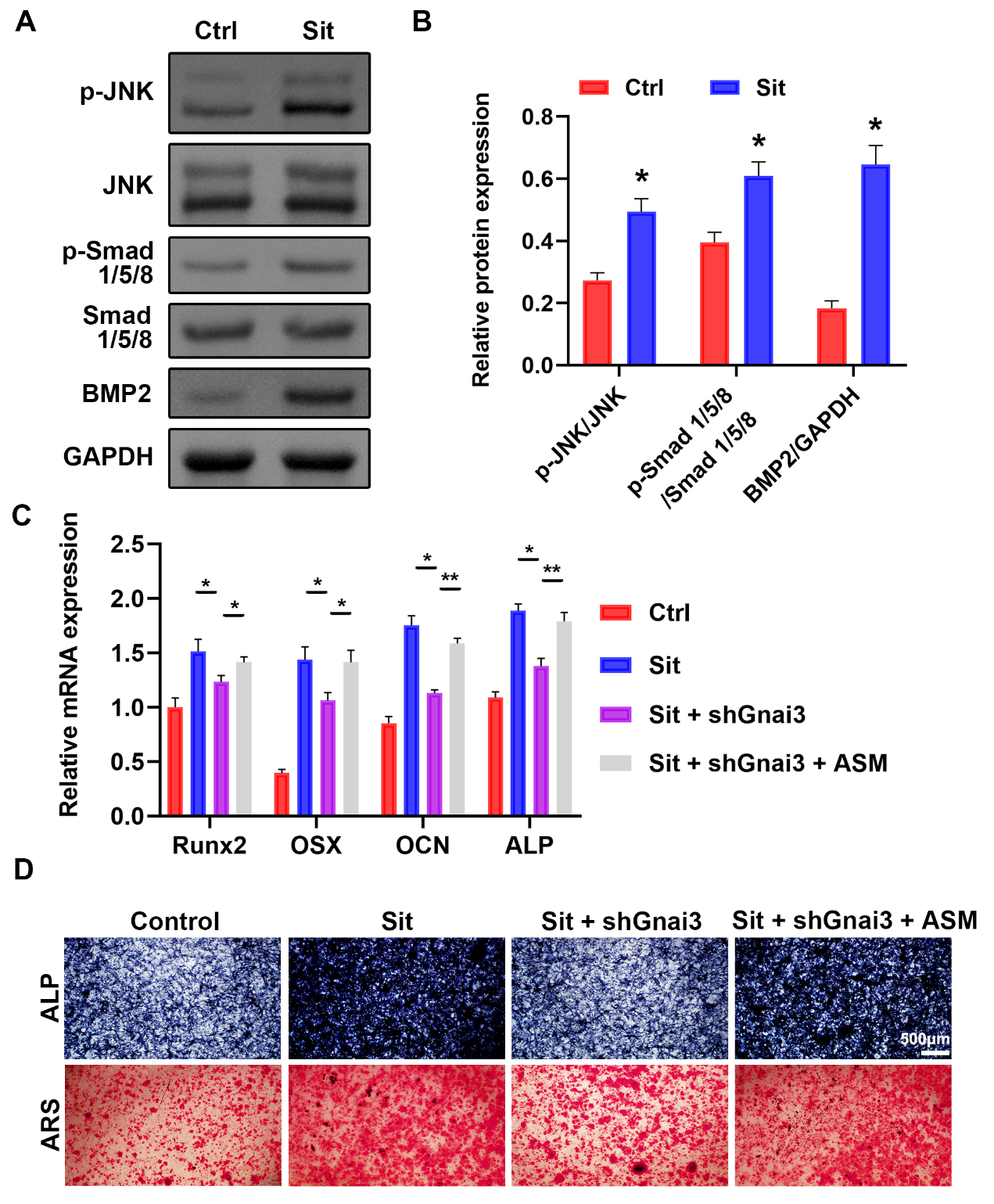
**

**Figure. S3. Sitogluside positively regulates osteogenic differentiation and via JNK signal pathway. A.** The protein expression of p-JNK, BMP2 and p-Smad levels detected by Western blotting. **B.** Quantitative analysis of p-JNK, BMP2 and p-Smad. **C**. qRT-PCR assay carried out for osteogenic differentiation marker genes in Ctrl, Sit, Sit+shGnai3 and Sit+shRNA (shGnai3)+ anisomycin (ASM, a p-JNK activator) groups. **D**. ALP and ARS staining in Ctrl, Sit, Sit+shGnai3 and Sit+shGnai3+ASM groups.
